# A subgroup of microRNAs defines PTEN-deficient, triple-negative breast cancer patients with poorest prognosis and alterations in RB1, MYC, and Wnt signaling

**DOI:** 10.1186/s13058-019-1098-z

**Published:** 2019-01-31

**Authors:** Dong-Yu Wang, Deena M. A. Gendoo, Yaacov Ben-David, James R. Woodgett, Eldad Zacksenhaus

**Affiliations:** 1 Toronto General Research Institute - University Health Network, 67 College Street, Rm. 407, Toronto, Ontario M5G 2M1 Canada; 2 Centre for Computational Biology, Institute of Cancer and Genomic Sciences, University of Birmingham, Birmingham, UK; 3 The Key laboratory of Chemistry for Natural Products of Guizhou Province and Chinese Academy of Sciences, Guiyang, 550014 Guizhou China; 4 State Key Laboratory for Functions and Applications of Medicinal Plants, Guizhou Medical University, Guiyang, 550025 China; 5 Lunenfeld-Tanenbaum Research Institute, Sinai Health System, 600 University Avenue, Toronto, ON Canada; 6 Department of Medicine, University of Toronto, Toronto, Ontario Canada

**Keywords:** TNBC, Prognosis, microRNA, PTEN, RB1, TP53, WNT, MYC, PI3K, Therapy

## Abstract

**Background:**

Triple-negative breast cancer (TNBC) represents a heterogeneous group of ER- and HER2-negative tumors with poor clinical outcome. We recently reported that Pten-loss cooperates with low expression of microRNA-145 to induce aggressive TNBC-like lesions in mice. To systematically identify microRNAs that cooperate with PTEN-loss to induce aggressive human BC, we screened for miRNAs whose expression correlated with PTEN mRNA levels and determined the prognostic power of each PTEN-miRNA pair alone and in combination with other miRs.

**Methods:**

Publically available data sets with mRNA, microRNA, genomics, and clinical outcome were interrogated to identify miRs that correlate with PTEN expression and predict poor clinical outcome. Alterations in genomic landscape and signaling pathways were identified in most aggressive TNBC subgroups. Connectivity mapping was used to predict response to therapy.

**Results:**

In TNBC, PTEN loss cooperated with reduced expression of hsa-miR-4324, hsa-miR-125b, hsa-miR-381, hsa-miR-145, and has-miR136, all previously implicated in metastasis, to predict poor prognosis. A subgroup of TNBC patients with PTEN-low and reduced expression of four or five of these miRs exhibited the worst clinical outcome relative to other TNBCs (hazard ratio (HR) = 3.91; *P* < 0.0001), and this was validated on an independent cohort (HR = 4.42; *P* = 0.0003). The PTEN-low/miR-low subgroup showed distinct oncogenic alterations as well as TP53 mutation, high RB1-loss signature and high MYC, PI3K, and β-catenin signaling. This lethal subgroup almost completely overlapped with TNBC patients selected on the basis of Pten-low and RB1 signature loss or β-catenin signaling-high. Connectivity mapping predicted response to inhibitors of the PI3K pathway.

**Conclusions:**

This analysis identified microRNAs that define a subclass of highly lethal TNBCs that should be prioritized for aggressive therapy.

**Electronic supplementary material:**

The online version of this article (10.1186/s13058-019-1098-z) contains supplementary material, which is available to authorized users.

## Background

Triple-negative breast cancers (TNBCs) are highly heterogeneous with certain tumors progressing to incurable metastatic disease. Pathologically, they are classified as estrogen receptor alpha (ERα)-negative, progesterone receptor-negative, and HER2/ERBB2/NEU-negative lesions [1]. No targeted therapy is currently available for TNBC, and patients are treated with aggressive chemotherapy. A recent phase III trial shows excellent synergy between T cell immune-checkpoint blockade therapy and cytotoxic chemotherapy in metastatic TNBC [2]. Although this treatment extended life span, patients succumbed to their disease. At the molecular level, TNBCs comprise basal-like and claudin-low/mesenchymal-like tumors and other subtypes [1, 3]. Moreover, within each subtype, mRNA-based expression signatures identify patients at high risk (reviewed in [4]). Alongside mRNA, microRNAs are also used to classify human cancer [5, 6], and integrated mRNA-miRNA signatures have been deployed [7]. Stratification of TNBCs and identification of patients with extremely poor clinical outcome are important to prioritize patients for aggressive treatment and identify new and personalized therapeutic avenues.

Disruption in RB1, PTEN, and TP53 occurs frequently in sporadic TNBC [8–10]. These three tumor suppressors are also the most frequent drivers of metastasis in diverse types of solid human cancers [11]. Thus, understanding the impact of these tumor suppressors on clinical outcome is informative not only for BC but also for other malignancies.

We recently demonstrated that inactivation of Pten in the mouse mammary gland induces mostly benign mammary tumors that fail to sprout secondary tumors following orthotopic transplantation into recipient mice. However, a rare group of Pten-deficient tumors with features of basal-like BC was efficiently transplantable [12]. These transplantable tumors exhibited low expression of microRNA-145, which was further demonstrated to functionally cooperate with Pten loss to promote tumorigenesis. These observations raised the question of whether in human BC and particularly in TNBC, PTEN-deficiency cooperates solely with miR-145 loss, with other or with additional microRNAs to define an aggressive subgroup of TNBCs. Here, we employed a systematic approach to identify microRNAs that cooperate with PTEN loss to predict poor clinical outcome. We identified a group of miRs comprising hsa-miR-145, hsa-miR-4324, hsa-miR-125b, hsa-miR-381, and has-miR136, the expression of which is lost together with PTEN in highly aggressive TNBC. These PTEN-low/miR-low TNBCs exhibit TP53 mutation, loss of RB1 signature, high signaling of MYC, WNT and PI3K pathways, and a distinct profile of predicted drug response. This cohort of patients should be prioritized for precision therapy.

## Methods

### Datasets selection and data processing

To identify correlated PTEN–miRNA expression pairs in BC, public datasets with both matched mRNA and miRNA data, immunohistochemical data for ER, PR, and HER2, as well follow-up survival tracking were used in this study. A European Genome-phenome Archive (https://www.ebi.ac.uk/ega/home), EGAS00000000122 [5, 13], containing 205 TNBCs and a total of 1302 BC patients with matched mRNA (EGAD00010000434) and miRNA (EGAD00010000438) data, was selected as a training cohort. When a particular gene was not available in EGAD00010000434, its mRNA expression was obtained from EGAS00000000083^7^, in which 1292 BC patients (205 TNBC) overlapped with EGAD00010000434. An NCBI Gene Expression Omnibus (https://www.ncbi.nlm.nih.gov/geo) Super Series GSE22220 [14] containing 44 TNBCs in a total of 207 BC samples with matched mRNA (GSE22219) and miRNA (GSE22216) data was used as a validation cohort. Downloaded mRNA and miRNA data with normalized Log2 format were transformed to a median center format for subsequent analysis. EGA study EGAS00001001753 [15] with copy number alteration (CNA) data on 1286 BCs, including 205 TNBCs, and gene mutation data of 1194 BC with related 185 TNBCs was used to identify genomic alterations of PTEN/miRNA tumors (Additional file 1: Figure S1).

### Subgroups and correlations

In the training cohort, 1302 BC samples were randomly subdivided into two BC subgroups A and B. And along with the subdivision, the 205 TNBCs of the 1302 BC were divided into two TNBC subgroups A and B as well, generating six subgroups of BC and six subgroups of TNBCs (Additional file 1: Figure S1 for details). Together with the 1302 BC and 205 TNBCs, a total of 14 groups were used to compare expression of PTEN and miRNAs in the training cohort. To identify the most correlated pairs, a Pearson correlation was performed between PTEN mRNA expression levels and all 853 miRNAs in each of the 14 subgroups. Rankings of the most positive or negative correlation coefficients were separately produced to evaluate the association between PTEN and each miRNA. Final sorting order of the association was determined by the average correlation ranking among all BC or TNBC subgroups.

### PTEN/miRNA co-expression profiling

For PTEN-low, we used twofold below median as a cut-off. For miRNAs, we optimized cut-offs using miR-low vs. miR-high for every single miRNA and then used the same cut-off for other analysis such as PTEN-low/miR-low vs others using Kaplan-Meier survival analysis in the training and validation cohorts.

### PTEN/miRNAs profiling by GSEA, mutations and CNVs, and CRNDE expression

To verify the connection between PTEN/miRNAs profiling and pathway activity of oncogenes and tumor suppressor genes, their relationship were evaluated in two ways: First, the activities of 18 oncogenic and tumor suppressor genes pathway signatures in microarray-based gene expression [16], plus a breast cancer RB1-loss signature [17], were estimated by using mRNA expression data. Second, a gene set contained 74 protein-coding cancer genes was obtained from a large-scale somatic mutation research in breast cancers [10] and used to distinguish the alteration of breast cancer genes by mRNA expression, copy number alterations, and gene mutations.

#### Identification of differentially expressed genes in connectivity map analysis

Gene expression patterns of 11 samples from subgroup “a” (Pten-low/miR-low tumors) were compared against the remaining 194 TNBC samples to identify differentially expressed genes. Log-normalized gene expression values were parsed in R (version 3.5.0), and differential expression was conducted using the *limma* package (version 3.36.2) [18] in R. A linear model per gene (*n* = 13,583 genes) was fit against the design matrix of the microarray experiment using the *lmfit* function, followed by an empirical Bayes adjustment using the *eBayes* function to generate several statistics for differential expression (t-stat, log-odds ratio). Final annotations and multiple-testing correction (FDR) adjustment was taken using the *topTable* function. Differentially expressed genes were considered based on FDR < 0.05.

#### Drug perturbation signatures

Transcriptional profiles of cancer cell lines treated with drugs as part of the BROAD Connectivity map initiative (CMAP; *n* = 1309 drugs) were obtained using the PharamcoGx package (version 1.10.0) [19] in R. Pre-computed drug perturbation signatures for CMAP were available for 1288 drugs and used in the downstream analysis. These signatures signify the drug concentration effect on the transcriptional state of the cell and were used to identify genes whose expression is perturbed by the drug treatment. Details of the linear regression model used to compute these signatures have been described [19].

#### Repurposing of CMAP drugs against the TNBC subgroup

Drug repurposing of CMAP drugs for the TNBC subgroup was conducted on the genes common to the TNBC gene-list and the CMAP perturbation signatures. TNBC “signatures” specific to the TNBC subgroup were chosen by selecting equal numbers of significant (FDR < 0.05) up- and downregulated differentially expressed genes. A total of four signature sizes were tested, spanning 100, 150, 200, and 298 genes, respectively. These TNBC signatures were compared against drug perturbation signatures from CMAP to identify drugs that could reverse the TNBC signature (i.e., could be a potential therapeutic drug for the TNBC subgroup).

Connectivity scores between CMAP drug perturbation signatures and each of the four TNBC signatures were computed using the *connectivityScore* function of the PharmacoGx package. Connectivity scores were computed once using the Gene Set Enrichment Analysis (GSEA) method (based on the KS statistic), and once using the Genome-Wide Connectivity (GWC) method (based on the weighted spearman statistic). Drugs were ranked by their connectivity score and associated *p* value, with more negative connectivity scores indicating an ability for a given drug to reverse the TNBC signature. Top hits were considered for drugs with connectivity scores below − 0.5 across all GSEA-based analyses and across all GWC-based analyses.

Rendering of connectivity scores across the four TNBC signatures was performed using the *qqman* package (version 0.1.4) in R. Top drug hits that were common between GSEA and GWC analyses, across each of the TNBC signatures tested, were identified using the *VennDiagram* package (version 1.6.17) in R.

### Additional statistical analysis

Prism 6 Software (GraphPad Software, La Jolla, CA, USA) was used for statistical analysis. Pearson correlation was used to evaluate associations between expression of PTEN mRNA and each miRNA in different subgroups. *T* test and ANOVA were used to calculate differences in gene expression, pathway activity, signature, CNA, and mutation status between different profiling groups. Kaplan-Meier survival analysis was used to compare survival curves, and log-rank (Mantel-Cox) test to calculate *p* values and hazard ratios (HR). All CMAP analyses have been conducted using the R statistical software (version 3.5.0) (https://www.r-project.org/); listed software dependencies are available on Bioconductor (BioC) or the Comprehensive Repository R Archive Network (CRAN).

## Results

### Identification of microRNAs whose expression correlates with Pten loss in breast cancers of all subtypes or in TNBC

**Fig. 1:**
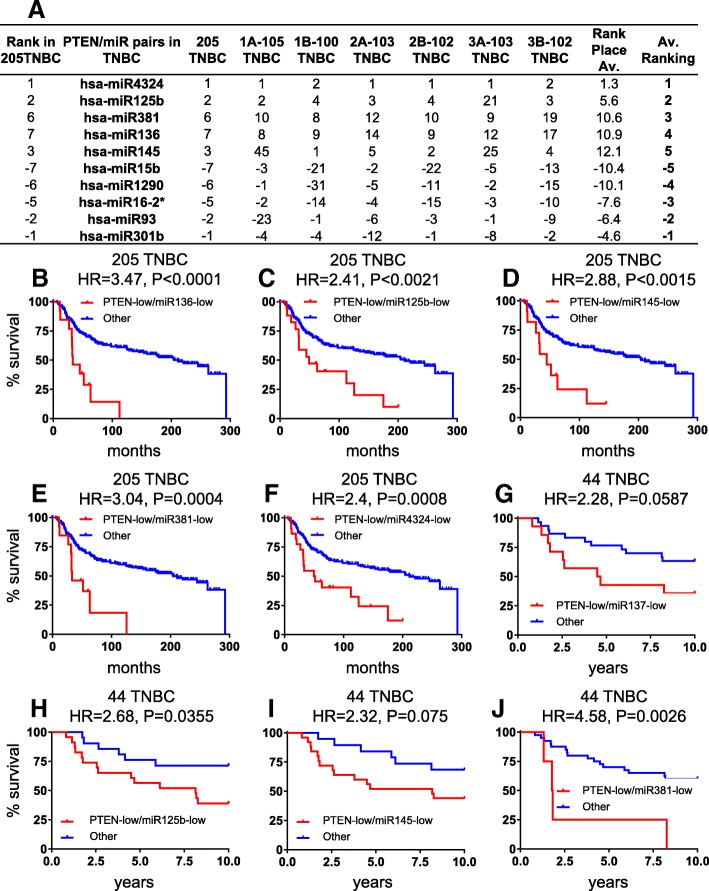
Identification of microRNAs whose expression levels most strongly correlate with Pten-low expression in TNBC. **a** Correlation ranking of top miRNAs with PTEN expression in TNBC. **b–f** Kaplan-Meier curves for top five positively correlated PTEN-miRNA pairs in 205 TNBC samples in the training cohort. **g**–**j** Kaplan-Meier curves for four positively correlated PTEN-miRNA pairs available in a 44 TNBC validation cohort

To identify microRNAs with expression that correlates with PTEN loss in BC, we used a study design and datasets depicted in Additionalfile 1: Figure S1A. The EGAS00000000122 dataset includes 1302 BC samples of which 205 (15.7%) are triple-negative tumors with mRNA and miRNA expression as well as clinical data. As low expression of PTEN mRNA is a strong predictor of its loss, we used low PTEN expression (twofold below median as a cut-off) to deduce the status of PTEN as previously described [12, 20]. Of the 205 TNBCs, 31 were designated PTEN-low by this criterion. Expression of each of the 853 miRNAs in the database was then correlated with PTEN expression. miRNAs that either positively or negatively correlated with low PTEN mRNA levels in all 1302 BC samples or in the 205 TNBC samples were identified (Additional file 2: Table S1A). As a first step toward verifying these miRNAs, we randomly divided the 1302 BC samples into three subgroups of ~ 652–653 samples and ranked the correlation of the miRNAs and PTEN expression in each (Additional file 2: Table S1B). Kaplan-Meier curves for all 1302 patients or for two of the three subgroups, classified by PAM50, reveal similar kinetics of over-all survival (Additional file 1: Figure S1B). Likewise, the 205 TNBC samples were divided into three subgroups of 100–105 and the correlation of miRNAs and PTEN expression ranked (Additional file 2: Table S1). We then averaged the ranking of each miRNA in the three sets of cohorts (Fig. 1a; Additional file 2: Table S2). In BC, we identified expression of hsa-miR-497, hsa-miR-4324, hsa-let-7c, hsa-miR-199a-5p, and hsa-miR-195 to consistently and positively correlate with Pten-loss, whereas phsa-miR-13535, hsa-miR-106b, hsa-miR-18a, hsa-miR-18b, and hsa-miR-93 to negatively correlate with low PTEN expression (Additional file 1: Figure S1C; Additional file 2: Table S1C). In TNBC, hsa-miR-4324, hsa-miR-125b, hsa-miR-381, hsa-miR-136, and hsa-miR-145 correlated positively, and hsa-miR-15b, hsa-miR-1290, hsa-miR-16-2*, hsa-miR-93, and hsa-miR-301b correlated negatively with PTEN-deficiency. Thus, hsa-miR-4324 scored as a positive correlate in both BC and TNBC, respectively, whereas hsa-miR-93 scored as a negative correlate in both BC and TNBC. The eight other miRs in each group were unique to either BC or TNBC.

### Expression of hsa-miR-4324, hsa-miR-125b, hsa-miR-381, hsa-miR-145, and has-miR136 cooperates with PTEN-deficiency to predict poor clinical outcome

**Fig. 2:**
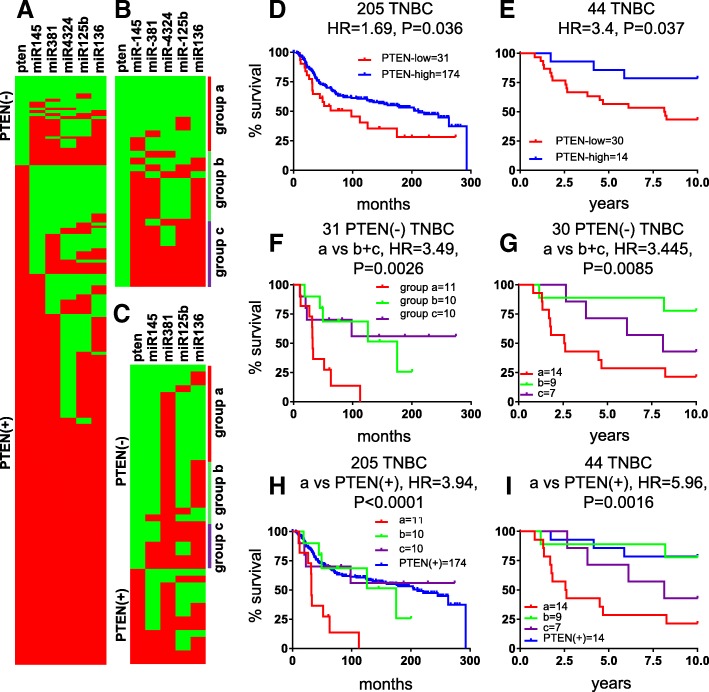
Identification of a PTEN-low/miR-low subgroup of TNBC with exceedingly poor prognosis. Heatmaps of PTEN and five most positively correlated miRNAs in 205 TNBC (**a**) and in 31 PTEN-deficient (−) TNBC (**b**) from training cohort and top four miRNAs in 44 TNBC (**c**) from the validation cohort. Map colors: green, low expression; red, high expression. Kaplan-Meier survival analysis on PTEN expression in training 205 TNBC (**d**) and validation 44 TNBC (**e**). **f**, **g** Kaplan-Meier survival analysis on PTEN-low/miR-low (group “a”) in 31 PTEN(−) TNBC from training cohort or 30 PTEN(−) TNBC from validation cohort. **h**, **i** Kaplan-Meier survival analysis on 205 TNBC or 44 TNBC for group “a” versus the remaining tumors

As PTEN is lost primarily in TNBC, we focused our attention on this aggressive subtype. First, we determined the hazard ratio (HR) for each of the ten PTEN-low-miRNA pairs in the training 205 TNBC cohort (Additional file 2: Table S3). Each of the five positive-correlated miRNAs, which were expressed at low levels together with PTEN, showed poor prognosis in Kaplan-Meier survival analysis and robust HR when combined with PTEN-loss. HR ranged from 3.471 (*P* < 0.0001) for hsa-miR-136 to 2.406 (*P* = 0.0008) for hsa-miR-4324 (Fig. 1b–f), compared with HR = 1.692 (*p* = 0.0358) for PTEN-low alone (Fig. 2d). In contrast, all five miRNAs with negative correlation with PTEN mRNA showed HR below 2, and only two miRs (hsa-miR-93 and hsa-miR-301b) yielded significant results when comparing PTEN-low/miR-high to all other TNBC (Additional file 2: Table S3).

Next, we validated the results on an independent cohort of 44 TNBCs (Additional file 1: Figure S1). Of the five positively correlated miRNAs, no miRNA data were available for hsa-miR-4324, but the other miRNAs either gave significant HRs (hsa-miR-125b and hsa-miR-381) or showed a trend toward significance (hsa-miR-136 and hsa-miR-145), possibly reflecting the relatively small number of patients in this cohort (Fig. 1g–j). For negatively correlated miRs, there was only expression data for hsa-miR-93 and this showed low HR and no significance (Additional file 2: Table S3). For subsequent studies, we therefore focused on the five positively correlating miRNAs: hsa-miR-4324, hsa-miR-125b, hsa-miR-381, hsa-miR-136, and hsa-miR-145.

### TNBCs with PTEN-low and low expression of 4 or 5 of hsa-miR-4324, hsa-miR-125b, hsa-miR-381, hsa-miR-145, or has-miR136 exhibit extremely poor clinical outcome

Inspection of a binary heat map of PTEN-deficient TNBCs revealed a subgroup of patients with low expression of hsa-miR-4324, hsa-miR-125b, hsa-miR-381, hsa-miR-136, and/or hsa-miR-145 (Fig. 2a, b). To provide some flexibility, we combined PTEN-deficient patient tumors with low expression of either four or all five miRNAs as group “a.” We designated group “c” as patient tumors with high expression of all five or four of these miRNAs, and group “b” as the remaining tumors. Kaplan-Meier analysis revealed that the PTEN-low/miR-low patients (group “a”) had significantly worse prognosis compared to groups “b” and “c” with HR = 3.91 (*P* < 0.0001; Fig. 2f, h). The PTEN-low/miR-low TNBC patients also had poor clinical outcome in the validation cohort relative to the other patients (HR = 4.42; *P* = 0.0003; Fig. 2c, e, g, i). All five miRNAs must be considered to select this subpopulation of aggressive TNBC. These results reveal the existence of a subgroup of TNBC patients, defined on the basis of PTEN-low/miR-low status, that exhibit a particularly poor prognosis that should be identified and urgently treated.

### Alterations in genomic and mRNA expression in PTEN-loss/miR-low TNBCs

**Fig. 3:**
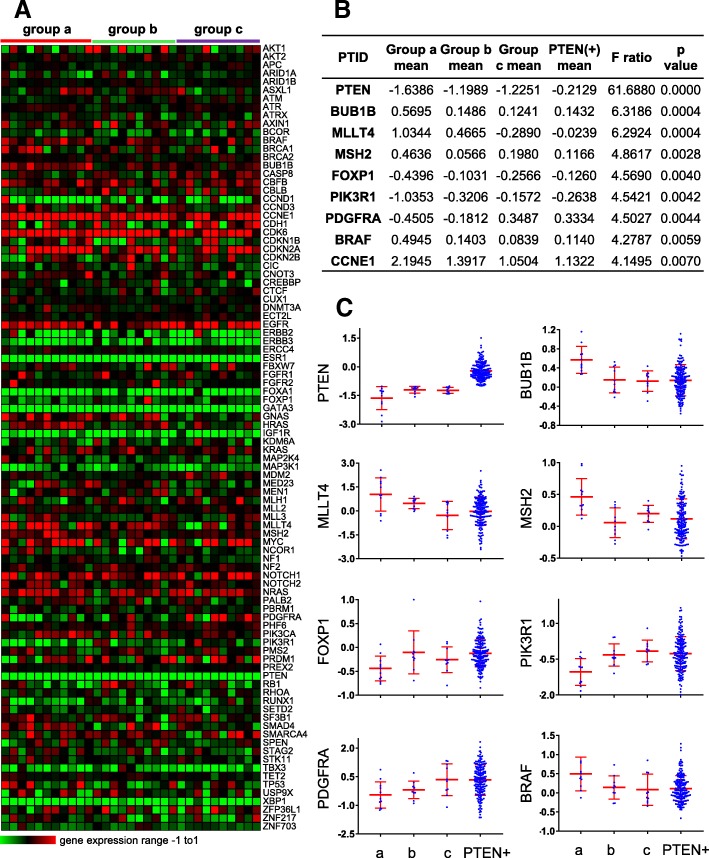
Expression analysis of frequently altered breast cancer genes in the PTEN-low/miR-low subgroup of TNBC. Heatmap for expression of 93 cancer genes in 31 PTEN(−) TNBC (**a**). ANOVA analysis of genes with significantly altered expression in PTEN-low/miR-low subgroup “a,” subgroups “b” and “c” or PTEN(+) TNBC (**b**). Scatter plots of selected genes with differentially altered expression in the PTEN/miRs groups in 205 TNBCs (**c**)

We next sought to determine whether PTEN-low/miR-low TNBCs harbor common genomic alterations that may be useful diagnostically or therapeutically. To this end, we took advantage of genomic data on mutation and copy number alteration (CNA) as well as mRNA expression available for these cohorts. We specifically looked for alterations in 93 genes commonly lost in BC as compiled from exome and whole genome sequencing [8–10, 21–24]. CNA analysis revealed significant gains of DNMT3A, and, surprisingly, of the luminal marker GATA3, as well as deletions of PTEN in the PTEN-low/miR-low tumors (Additional file 1: Figure S2A, S3). These CNAs correlated with low mRNA expression of PTEN but not with high expression of DNMT3A or GATA3, the latter of which is expressed at low levels in all TNBCs (Fig. 3). Mutational analysis revealed common alterations only in p53 (Additional file 1: Figure S2B). While all PTEN-low/miR-low tumors had p53 mutations, two of each group “b” and group “c” tumors lacked p53 mutation. Our CNA analysis revealed that three of the latter four tumors show no gain of its E3 ligase HDM2 and presumably harbor p53 deletions instead [9]. Notably, p53 mutations may have dominant gain-of-function effects that promote metastasis (reviewed in [25]).

More revealing was mRNA expression analysis (Fig. 3). First, as expected, expression levels of several luminal markers such as ERα, GATA3, FOXA1, and XBP1 was low in all 31 TNBC samples. While expression of PTEN was invariably low, it was lowest in the PTEN-low/miR-low (group “a”) tumors (Fig. 3b, c). Expression of FOXP1 (Forkhead Box P1), PIK3R1 (phosphoinositide-3-kinase regulatory subunit 1), and PDGFRA (platelet derived growth factor receptor alpha), implicated in ER^+^ BC, was also the lowest in this group. In contrast, expression of BUB1B (BUB1 mitotic checkpoint serine/threonine kinase B), MLLT4 (a RAS target involved in cell–cell adhesions), MSH2 (DNA mismatch repair protein), BRAF (a kinase within the RAS-MAPK pathway), and CCNE1 (cyclin E; upstream of RB1) was highest in group “a” compared to groups “b,” “c” or PTEN-positive tumors.

### PTEN-low/miR-low TNBCs exhibit high RB1-loss signature and elevated MYC, PI3K, and β-catenin signaling

**Fig. 4:**
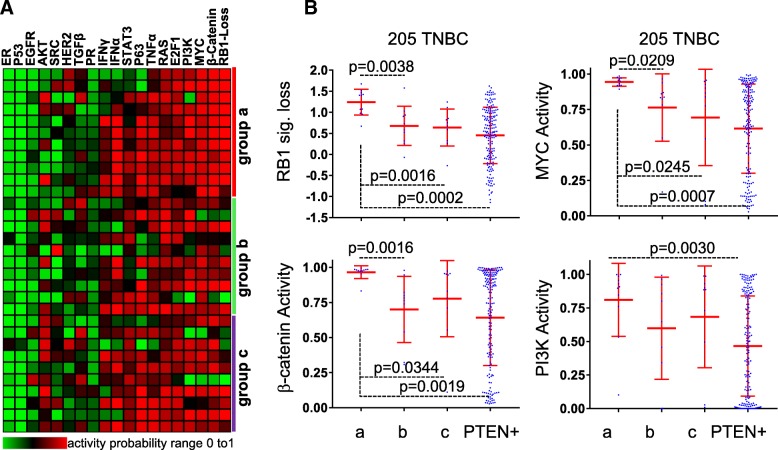
RB1 pathway loss and high β-catenin, WNT, and PI3K signaling in the PTEN-low/miR-low subgroup of TNBC. **a** Heatmap of probability of pathway activity in 31 PTEN(−) TNBCs from training cohort. **b** *t* test of probability of pathway activity in 205 TNBC from training cohort

A caveat of gene specific analysis is that while alterations in each specific gene along a given pathway may be infrequent, the whole pathway may be altered in each tumor through different alterations in pathway constituent genes; this can be missed when only small groups of samples are analyzed. We therefore performed pathway activity analysis to capture alterations affecting entire signaling pathways. We analyzed 18 signaling pathways using signatures developed by Gatza et al. [16], as well as a signature for RB1-loss [17, 26], in group “a” versus “b” plus “c.” Remarkably, the RB1-loss signature, PI3K, β-catenin and MYC (*P* < 0.01), and to a lesser extent E2F1 (*P* < 0.05), showed increased pathway activity in the PTEN-low/miR-low subgroup compared to the other PTEN-deficient TNBCs (Fig. 4a). Notably, EGFR pathway activity was low in all 31 PTEN-deficient TNBCs relative to PTEN(+) TNBC (*P* = 0.0084; Fig. 4a; Additional file 1: Figure S4), demonstrating again the diversity of these tumors.

**Fig. 5:**
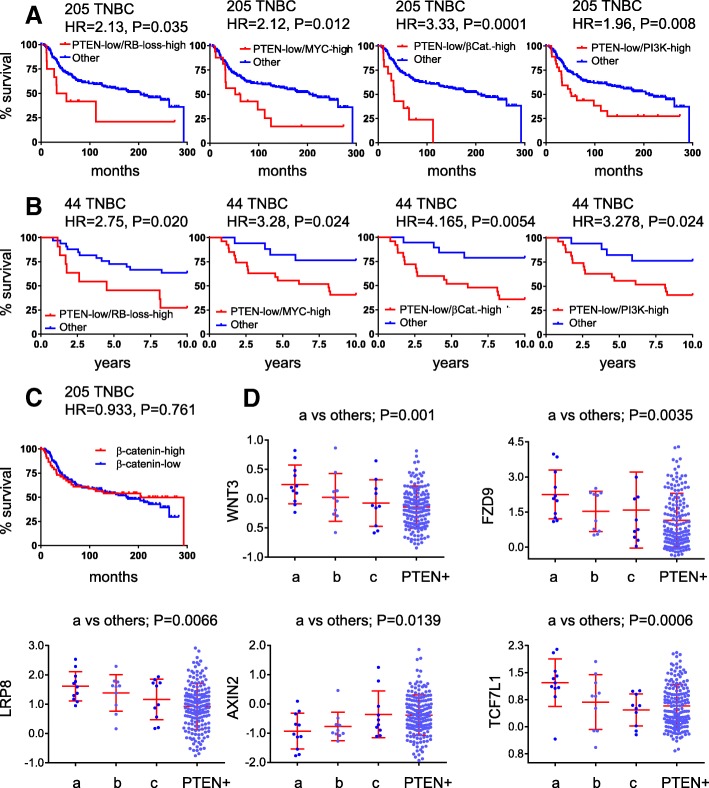
PTEN-low plus high Wnt/β-catenin and PI3K signaling or RB1 pathway loss identify TNBC patients with poor clinical outcome. **a**, **b** Kaplan-Meier survival curves for PTEN-low plus RB1 signature loss, or high MYC, β-catenin, or PI3K pathway activity in the 205 TNBC training or 44 TNBC validation cohorts. **c** High β-catenin pathway activity alone does not stratify TNBC into high and low clinical outcome. **d** Altered expression of genes on the Wnt/β-catenin pathway in PTEN-low/miR-low TNBC (subgroup “a”) compared to subgroup “b,” “c” or PTEN(+) TNBC in the 205 TNBC training cohort

In accordance with their TNBC status, all tumors exhibited low p53 and ER pathways, indicative of ER^−^ and p53-loss. RAS signaling was high but not significantly different among the 31 different PTEN-deficient TNBCs. Levels of RB1-loss signature, PI3K, β-catenin. and MYC pathway activities in each group of patients, and survival curves of PTEN-low/signature-high for each pathway in the two clinical cohorts are shown in Fig. 4b and Fig. 5a, b, respectively. The most striking cooperation was seen between Pten-low and β-catenin signaling-high (HR = 3.33, *P* < 0.0001 for the 205 TNBCs and HR = 4.165, *P* = 0.0054 for the 44 TNBC cohort). This cooperation is striking given that the HR for PTEN-low is 1.692 (Fig. 2d) and for high β-catenin signaling alone is insignificant (Fig. 5c).

### Landscape of genomic alterations in the WNT pathway in PTEN-low/miR-low and PTEN-low/β-catenin-pathway-high subgroups of TNBCs

The PTEN-loss/β-catenin-pathway-high group included 14 patients. Of these, 10 (of 11 patients) were from group “a,” one from group “b” and three from group “c.” To identify possible drivers of high β-catenin/WNT signaling in the PTEN-low/miR-low and PTEN-low/β-catenin-pathway-high subgroups of TNBCs, we analyzed alterations in mRNA, CNAs, and mutational landscapes in these tumors compared to all other lesions in the 205 TNBC database. Expression analysis of components of the canonical WNT signaling revealed significant increase in several genes including WNT3, FZD9, LRP8, and TCF7L1, as well as reduction in Axin2 in both the PTEN-low/miR-low and PTEN-low/β-catenin-pathway-high subgroups (Additional file 1: Figure S5 and S6). Notably, Axin2 acts to down-regulate Wnt signaling. Scatter plots of these genes are shown in Fig. 5d.

Mutational analysis of all Wnt pathway genes revealed no group “a” specific alterations (Additional file 1: Figure S7). A single APC mutation was found in group “b,” two in the PTEN(+) TNBC and none in group “a” or “c.” Likewise, no APC mutations were found in PTEN-low/β-catenin-pathway-high group but three were found in all “others.” No significant CNAs were found in these groups either. Thus, the major differences we could detect in Wnt signaling were at the level of mRNA.

Of the five microRNAs that are downregulated in group “a,” miR-136 has been implicated in Wnt signaling [27]. MiR-136 and other miRNAs such as miR-451 and miR-181a are known targets of the colorectal neoplasia differentially expressed (CRNDE) long non-coding RNA (lncRNA) [28]. However, although CRNDE expression is significantly higher in group “a” versus PTEN(+) TNBC, the highest expression of this lncRNA was found in Luminal B and HER2+ BC, and, importantly, there was no correlation between CRNDE and miR-136 expression in TNBC (Additional file 1: Figure S8 and S9). Although the mechanism by which Wnt signaling is induced in PTEN-low/miR-low TNBC remains to be elucidated, the level of activation of this pathway provides a therapeutic target for these aggressive tumors.

Finally, we asked whether the five miRNAs identified herein target genes on the MYC, β-catenin, and/or PI3K signaling pathways, which are elevated in subgroup “a.” To this end, miRNA targets mining was performed to search for verified interactions and predicted miRNA binding sites using an updated miRWalk platform version 3 (http://mirwalk.umm.uni-heidelberg.de) [29]. A total of 6327 candidate target genes with 19,287 miRNA binding sites was obtained with at least one of the five identified miRNAs. These targets overlapped widely between the five miRNAs (Additional file 1: Figure S10A). Among the aggregate 6327 candidate target genes, there were 223 targets that overlapped between the pathway activity training genes for PI3K, MYC or β-catenin pathways, determined as described by Gatza et al. [30] (Additional file 1: Figure S10B). By collecting mRNA microarray probes from both EGAD00010000434 and EGAS00000000083 datasets, expression of the 221 genes in the TNBC subgroups was determined. Top 20 targets with most differential expression within groups “a,” “b,” “c” and PTEN(+) TNBC were selected by ANOVA (*P* < 0.05; Additional file 1: Figure S10C). Six targets that overlapped between the five identified miRNAs and/or MYC, β-catenin and PI3K pathways more than once and with significantly higher expression (*t* test, *P* < 0.05) in groups “a” vs PTEN(+) TNBC are highlighted in panel c and their expression in groups “a,” “b,” “c” and PTEN(+) is shown in Additional file 1: Figure S10D. Thus, enhanced MYC, β-catenin and PI3K signaling observed in PTEN-low/miR-low TNBC is at least in part due to direct dysregulation of genes on these pathways in response to low expression of these miRNAs.

### Connectivity map analysis identifies distinct therapeutic targets for PTEN-low/miR-low TNBCs

**Fig. 6:**
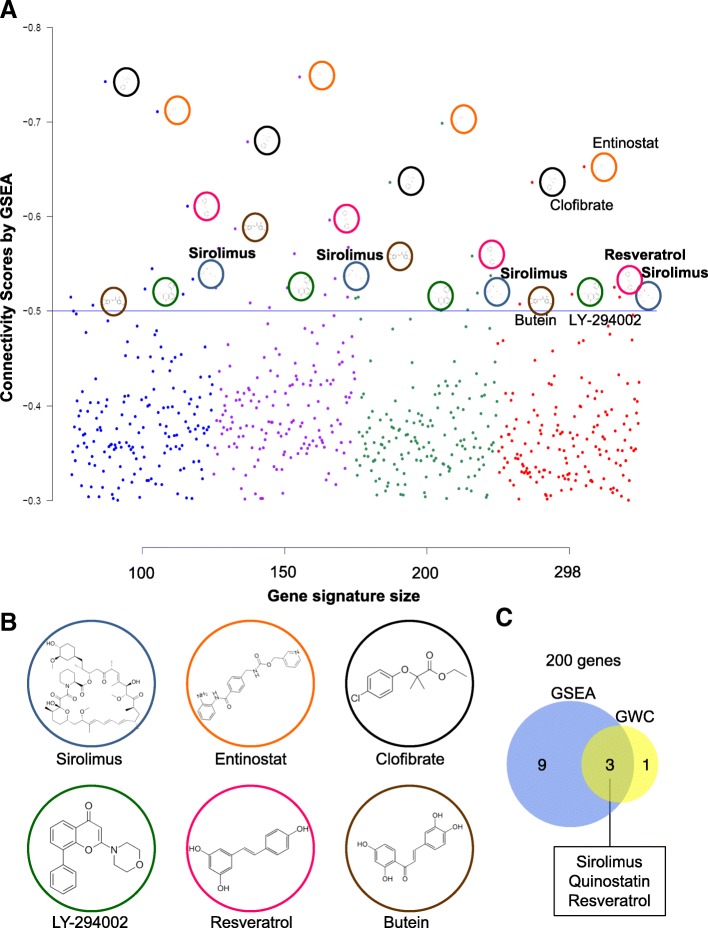
Connectivity map by GSEA identifies PI3K and other inhibitors for PTEN-low/miR-low subgroup of TNBC. **a** Connectivity scores (CS) of drug hits generated using the GSEA method and different sizes of the TNBC PTEN-low/miR-low subgroup of TNBC/ group “a” signature (four signature sizes). Each dot represents the connectivity score of a specific drug and colored to reflect the gene signature size used in the connectivity map analysis. Dots plotted represent drug hits that have a negative CS < (− 0.3) across all signature sizes. Dots above the CS line of − 0.5 indicate drugs that have a better ability to reverse the TNBC group “a” signature in the connectivity map analysis. **b** Structure of drugs that show consistent top hits (CS < − 0.5 across all gene signature sizes) by GSEA. **c** Venn diagram showing three drugs that appear in both GSEA and GWC analysis with 200 genes. Connectivity map analysis by GWC and additional Venn diagrams with different gene size are shown in Additional file 1: Figure S9 and 10

To independently determine whether PTEN-low/miR-low TNBCs may be therapeutically targeted, we calculated connectivity scores [31] via Gene Set Enrichment Analysis (GSEA) and Genome-Wide Connectivity (GWC) map as described [19, 32], comparing the 11 PTEN-low/miR-low TNBCs to the other 194 (205 minus 11) TNBC samples. In keeping with high PI3K pathway activity in these PTEN-deficient TNBCs, both methods identified antagonists of the PI3K/mTOR pathway: sirolimus (rapamycin, an mTOR inhibitor), quinostatin (a small-molecule inhibitor of class Ia PI3Ks), and LY294002 (inhibitor of phosphoinositide 3-kinases (PI3Ks), Fig. 6 and Additional file 1: Figure S11 and S12). In addition, both methods identified clofibrate, which depletes the levels of the lipoprotein VLDL and thereby promotes breast cancer through PI3K signaling [33], and resveratrol, which has pleiotropic effects including attenuation of PI3K signaling [34]. Thus, multiple drugs that directly or indirectly suppress PI3K signaling are predicted to kill this aggressive subgroup of TNBC.

## Discussion

We report the identification of a highly aggressive subgroup of TNBCs that express low level of PTEN mRNA together with low level of four or all five of the following microRNAs: hsa-miR-4324, hsa-miR-125b, hsa-miR-381, hsa-miR-145, and has-miR136. These microRNAs have been implicated in invasion and metastasis in breast cancer and other malignancies [12, 27, 35–37], suggesting that their low expression is a driver rather than a surrogate of poor prognosis. Whether these miRs represent primary oncogenic alterations or a reflection of upstream oncogenic events that suppress their expression is yet to be determined. The PTEN-low/miR-low TNBC subgroup exhibited significant hazard ratio of 3.91 and 4.42 in two independent cohorts compared to other TNBC samples. These tumors harbor TP53 mutations, RB1 loss and high MYC, WNT/β-catenin, and PI3K signaling activity relative to other PTEN-low tumors that do not express low levels of these miRNAs. Indeed, the five microRNAs target genes on the MYC, β-catenin, and PI3K pathways, which may explain at least in part their enhanced activity in this aggressive subgroup of TNBC. These lethal TNBCs overlap to a large extent with TNBC identified on the basis of PTEN-low expression plus RB1-signature loss, or plus high MYC, PI3K, or β-catenin signaling. These patients should be identified and prioritized for specific therapy. Our results point to possible therapeutic strategies for these patients including inhibitors of PI3K and Wnt signaling. These PTEN-deficient tumors also show high levels of RAS signaling and hence are expected to respond to RAS pathway antagonists such as MEK inhibitors [12]. In addition, all PTEN-low/miR-low TNBCs showed p53 mutations rather than deletions and may therefore respond well to drugs that induce degradation of mutant p53 or its conversion to a wild-type-like protein [38].

Generation of a preclinical mouse model for PTEN-low/miR-low TNBCs would facilitate the assessment of potential therapies for these aggressive tumors. However, this is a formidable task. Our observations that Wnt signaling is highly induced in these tumors, and that PTEN-low, β-catenin signaling-high identifies TNBCs with extremely poor prognosis suggest that a composite mouse model based on loss of Pten, activation of β-catenin, and mutation in p53 may exhibit aggressive/metastatic tumors that would mimic human PTEN-low/miR-low TNBC. β-catenin activation can be experimentally achieved by conditional expression of a transgene with exon 3 deletion ([39] and references therein). However, such β-catenin mutations do not occur in BC, but rather this pathway is induced by multiple upstream mechanisms to promote diverse types of cancer including TNBC [40, 41]. Our search for alterations in the WNT pathway in PTEN-low/miR-low (subgroup “a”) or in the PTEN-low/β-catenin signaling-high subgroups only revealed differences at the mRNA level such as over-expression of WNT3, FZD9, LRP8, and TCF7L1. Possibly, the trigger for Wnt signaling is provided by the unique stroma of TNBC [42, 43], other signaling pathways such as NFkB [44], or cooperation between different tumor clones [45]. Alternatively, other genomic and proteomic alterations such as in RNF43, ZNRF3, RSPO2, or RSPO3, not interrogated in our study, may drive β-catenin pathway activation in PTEN-low/miR-low tumors [46]. Notably, a recent study links intracellular pH to β-catenin stability [47], indicating that tumor microenvironment and post-translational modifications rather than oncogenic alterations may drive Wnt signaling in TNBC.

The observation that PTEN-low/miR-low TNBCs also express high level of RB1-loss signature is consistent with a recent report that these tumor suppressors together with p53 are frequently lost in diverse type of solid metastases [11]. Indeed, TNBC cell lines such as BT549 harbor mutations in all these tumor suppressors and are highly aggressive following transplantation into immune-deficient mice [20, 26]. We previously demonstrated strong cooperation between loss of Rb and p53 [48, 49], and between loss of Pten and p53 [20, 50] in mouse models. It would be thus of interest to generate triple Rb/Pten/p53-mutant tumors and study their metastatic behavior. We expect these tumors to be highly sensitive to PI3K inhibition. In addition, we recently identified CDC25 as a common therapeutic target for diverse TNBCs, including RB1/PTEN/P53-deficient TNBC [20], and demonstrated a strong synergy between CDC25 and PI3K/mTOR inhibitors even in tumors with intact PTEN expression. We therefore expect that PTEN-low/miR-low TNBCs identified herein, which show alterations in p53 and the RB1 pathway, to be highly sensitive to this combination therapy.

mRNA- and microRNA-based signatures from primary lesions and circulating tumor cells have been extensively used to predict clinical outcome, and some are in clinical use [35, 51–53]. We show herein that a PTEN-low/miR-low “signature” provides potent prognostication of TNBC. The approach we developed here, which can be simplified by appropriate algorithms, can be used to develop additional integrated mRNA-miRNA-based classifications to stratify cancer patients.

## Conclusions

We report the identification of a subclass of TNBCs with extremely poor prognosis that should be prioritized for aggressive therapy. These lethal TNBCs express low levels of five microRNAs, defined in this study, as well as alterations in PI3K/PTEN, RB1, MYC, and WNT signaling. These features plus an in silico drug prediction analysis point to a few potential therapeutic targets such as PI3K and Wnt signaling. Our analysis also provides a rationale for analyzing cooperating oncogenic driver-miRNA combinations in diverse types of cancer.

## Additional files

Additional file 1: **Figure S1.** Overview of breast cancer (BC) datasets, cohorts and groups used in this project, and subgrouping analysis. (A) The 1302 BC dataset, which includes 205 triple-negative breast cancers (TNBC) with matched mRNA and miRNA data from EGAS00000000122, was used as training cohort. Six subgroups of all BC or TNBC were randomly divided to correlate expression of PTEN and miRNAs. The 207 BC dataset, which contained 44 TNBC with matched mRNA and miRNA data from GSE22220, was used as validation cohort. 205 related TNBC with copy number alteration (CNA) data and 185 related TNBC with gene mutation data from EGAS00001001753 were used to confirm genomic changes of PTEN-miRNA co-expression profile. (B) Kaplan-Meier survival analysis on PAM50 classification of all 1302 breast cancers (BC), and examples of subgroups 2A-651 BC and 2B-651 BC. (C) Heatmap of correlation coefficient (r) between PTEN and miRNAs for most positive or negative correlation in BC (left) or TNBC (right). **Figure S2.** DNA sequence variations in TNBC subgroups. (A) Heatmaps of Copy Number Alteration (CNA) of 93 protein-coding cancer genes among the different subgroups in 31 PTEN(-) TNBC. (B) Mutational landscape of 74 genes that have at least one mutated gene among the TNBC subgroups in 28 PTEN(-) TNBC.. **Figure S3.** Significant changes in copy number alterations (CNA) in protein-coding cancer genes among TNBC subgroups. CNA of total gain (1 + 2) and loss (-1 + -2) in TNBC subgroups and CNA changes of CUX1, DNMT3A, GATA3, MMLLT4, MYC, PBRM1, PTEN and ZNF217. **Figure S4.** Low EGFR pathway activity in PTEN-deficient TNBC including subgroup ‘a’ as compared to PTEN+ tumors. **Figure S5.** mRNA expression and CNA of Wnt/β-catenin signaling related genes in PTEN-low/miRs-low (subgroup ‘a’) TNBC versus other TNBC. **Figure S6.** mRNA expression and CNA of Wnt/β-catenin signaling related genes in PTEN(-)/β-catenin(+) TNBC versus other TNBC. **Figure S7.** Mutation in PTEN/β-catenin(+) TNBC versus other TNBC. 173 gene mutation data were compared and 135 genes with at least one mutation are shown in order of the number of mutated genes. **Figure S8.** CRNDE mRNA expression level and distribution in 1292 BC in EGAS00000000083. (A) Expression level of CRNDE mRNA in high (> 1), medium (1 to 0) and low (< 0) was tested by Log-rank test and revealed no significant difference. CRNDE distribution of mRNA expression was compared in TNBC subgroups (B) and PAM50 subtypes (C) by ANOVA and t-test. **Figure S9.** Correlation between CRNDE expression and target miRNAs in TNBC subgroups and PAM50 subtypes. Pearson correlation was preformed between CRNDE and its target miRNAs miR-136 and miR-451 in TNBC subgroups and PAM50 subtypes. CRNDE targets miRNAs miR-384 and miR-181a-5p are not available in EGAD00010000438 miRNA dataset; four miR-181a-related miRNAs were tested here. **Figure S10.** Target miRNAs are predicted to regulate MYC, β-Catenin and PI3K signalling pathways. (A) Predicted target genes and overlap between the five identified miRNAs using miRWalk3 miRNAs target mining tool. (B) Detected target genes overlap with MYC, β-Catenin 3 and PI3K pathway activity genes. (C) mRNA expression of top 20 detected target genes on the MYC, β-Catenin and PI3K pathways that are regulated by the five identified miRNAs. (D) mRNA expression of six detected targets of the five miRNAs and/or MYC, β-Catenin and PI3K pathway training genes that appear more than once in panel C. **Figure S11.** Connectivity map by GWC identifies PI3K and other drugs for PTEN-low/miRs-low subgroup of TNBC. Connectivity scores (CS) of drug hits generated using the GSEA method and different sizes of the PTEN-low/miRs-low TNBC (group ‘a’; 4 signature sizes). Each dot represents the connectivity score of a specific drug, and colors reflect gene signature size used in the connectivity map analysis. Dots plotted represent drug hits that have a negative CS < (-0.3) across all signature sizes. Dots above the CS line of -0.5, indicate drugs that have a better ability to reverse the TNBC group ‘a’ signature in the connectivity map analysis. No drugs had score <-0.5 across all 4 runs. Thus, for this analysis, the stringency cut-off was set at <-0.45). **Figure S12.** Overlap between drug hits using GSEA and GWC connectivity scoring metrics. The number of drug hits is based on group ‘a’ TNBC gene signature size tested, with CS <-0.5. Common drugs identified by both methods in each analysis are highlighted. For 200 gene size, see Fig. 6c. (PPTX 2216 kb)

Additional file 2: **Table S1.** Ranking of correlation coefficients in top 40 pairs of PTEN vs. miRNAs from each of the 14 subgroups. **Table S2.** Average ranking of correlation coefficients in top 40 miR pairs on 7 BC subgroups and 7 TNBC subgroups. **Table S3.** Log-rank test of average-ranked top 20 PTEN/miRNAs pairs in all BC and TNBC on EGAS00000000122 and GSE22220 datasets. (XLSX 33 kb)

## Abbreviations

BC: Breast cancer
CRNDE: Colorectal Neoplasia Differentially Expressed
ERα: Estrogen receptor alpha
GSEA: Gene Set Enrichment Analysis
GWC: Genome-Wide Connectivity
HER2: Human epidermal growth factor receptor 2
lncRNA: Long non-coding RNA
miR: microRNA
PTEN: Phosphatase and Tensin homolog deleted in chromosome 10
RB1: Retinoblastoma gene
TNBC: Triple-negative breast cancer
TP53: Tumor protein 53
