# Supplementary material for: A subgroup of microRNAs defines PTEN-deficient, triple-negative breast cancer patients with poorest prognosis and alterations in RB1, MYC, and Wnt signaling
Source: Breast Cancer Res. 2019 Jan 31;21:18. doi: 10.1186/s13058-019-1098-z (PMC6357448; doi:10.1186/s13058-019-1098-z)
Supplement: Supplementary file 1 — Figure S1. Overview of breast cancer (BC) datasets, cohorts and groups used in this project, and subgrouping analysis. (A) The 1302 BC dataset, which includes 205 triple-negative breast cancers (TNBC) with matched mRNA and miRNA data from EGAS00000000122, was used as training cohort. Six subgroups of all BC or TNBC were randomly divided to correlate expression of PTEN and miRNAs. The 207 BC dataset, which contained 44 TNBC with matched mRNA and miRNA data from GSE22220, was used as validation cohort. 205 related TNBC with copy number alteration (CNA) data and 185 related TNBC with gene mutation data from EGAS00001001753 were used to confirm genomic changes of PTEN-miRNA co-expression profile. (B) Kaplan-Meier survival analysis on PAM50 classification of all 1302 breast cancers (BC), and examples of subgroups 2A-651 BC and 2B-651 BC. (C) Heatmap of correlation coefficient (r) between PTEN and miRNAs for most positive or negative correlation in BC (left) or TNBC (right). Figure S2. DNA sequence variations in TNBC subgroups. (A) Heatmaps of Copy Number Alteration (CNA) of 93 protein-coding cancer genes among the different subgroups in 31 PTEN(-) TNBC. (B) Mutational landscape of 74 genes that have at least one mutated gene among the TNBC subgroups in 28 PTEN(-) TNBC.. Figure S3. Significant changes in copy number alterations (CNA) in protein-coding cancer genes among TNBC subgroups. CNA of total gain (1 + 2) and loss (-1 + -2) in TNBC subgroups and CNA changes of CUX1, DNMT3A, GATA3, MMLLT4, MYC, PBRM1, PTEN and ZNF217. Figure S4. Low EGFR pathway activity in PTEN-deficient TNBC including subgroup ‘a’ as compared to PTEN+ tumors. Figure S5. mRNA expression and CNA of Wnt/β-catenin signaling related genes in PTEN-low/miRs-low (subgroup ‘a’) TNBC versus other TNBC. Figure S6. mRNA expression and CNA of Wnt/β-catenin signaling related genes in PTEN(-)/β-catenin(+) TNBC versus other TNBC. Figure S7. Mutation in PTEN/β-catenin(+) TNBC versus other TNBC. 173 gene mutation da [file 13058_2019_1098_MOESM1_ESM.pptx]

## Slide 1
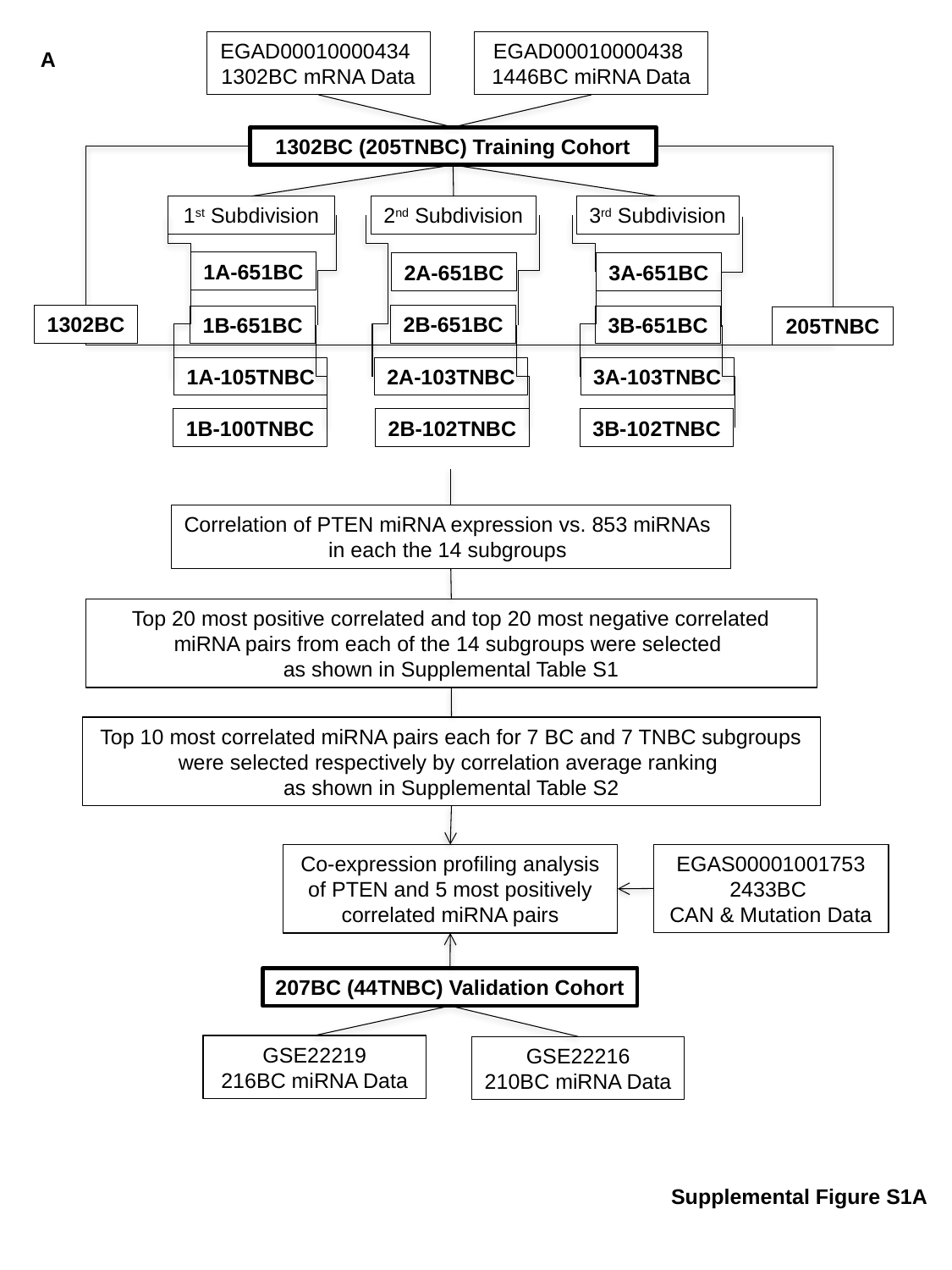

EGAD00010000434
1302BC mRNA Data
EGAD00010000438
1446BC miRNA Data
1302BC (205TNBC) Training Cohort
1st Subdivision
1A-651BC
1B-651BC
1A-105TNBC
1B-100TNBC
2nd Subdivision
2A-651BC
2B-651BC
2A-103TNBC
2B-102TNBC
3rd Subdivision
3A-651BC
1302BC
3B-651BC
205TNBC
3A-103TNBC
3B-102TNBC
Correlation of PTEN miRNA expression vs. 853 miRNAs
in each the 14 subgroups
Top 20 most positive correlated and top 20 most negative correlated miRNA pairs from each of the 14 subgroups were selected
as shown in Supplemental Table S1
Top 10 most correlated miRNA pairs each for 7 BC and 7 TNBC subgroups were selected respectively by correlation average ranking
as shown in Supplemental Table S2
EGAS00001001753
2433BC
CAN & Mutation Data
Co-expression profiling analysis of PTEN and 5 most positively correlated miRNA pairs
207BC (44TNBC) Validation Cohort
GSE22219
216BC miRNA Data
GSE22216
210BC miRNA Data
A
Supplemental Figure S1A

## Slide 2
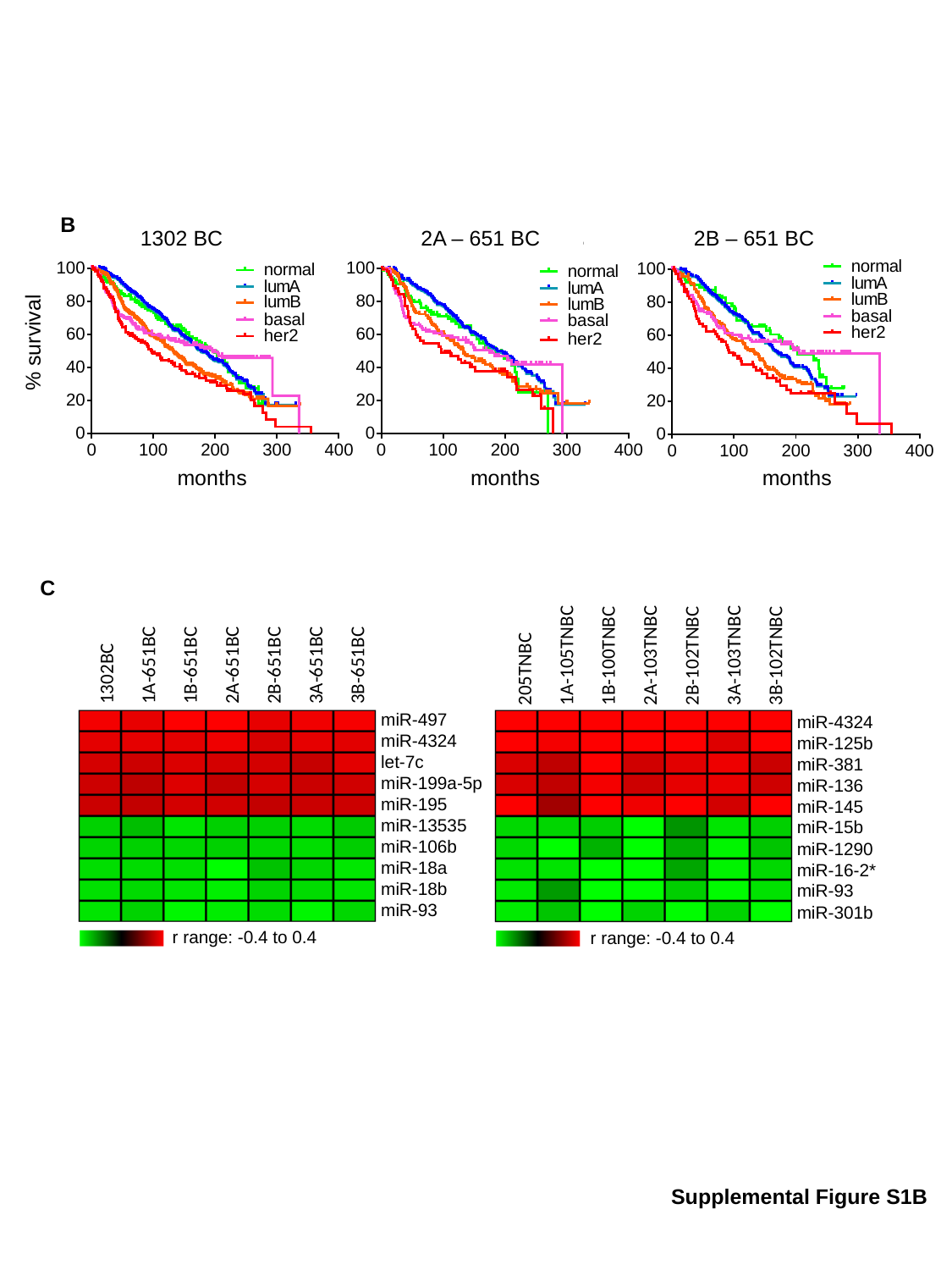

1302 BC
 2A – 651 BC
2B – 651 BC
B
 % survival
months
months
months
C
205TNBC
1A-105TNBC
1B-100TNBC
2A-103TNBC
2B-102TNBC
3A-103TNBC
3B-102TNBC
r range: -0.4 to 0.4
miR-4324
miR-125b
miR-381
miR-136
miR-145
miR-15b
miR-1290
miR-16-2*
miR-93
miR-301b
1302BC
1A-651BC
1B-651BC
2A-651BC
2B-651BC
3A-651BC
3B-651BC
r range: -0.4 to 0.4
miR-497
miR-4324
let-7c
miR-199a-5p
miR-195
miR-13535
miR-106b
miR-18a
miR-18b
miR-93
Supplemental Figure S1B

## Slide 3
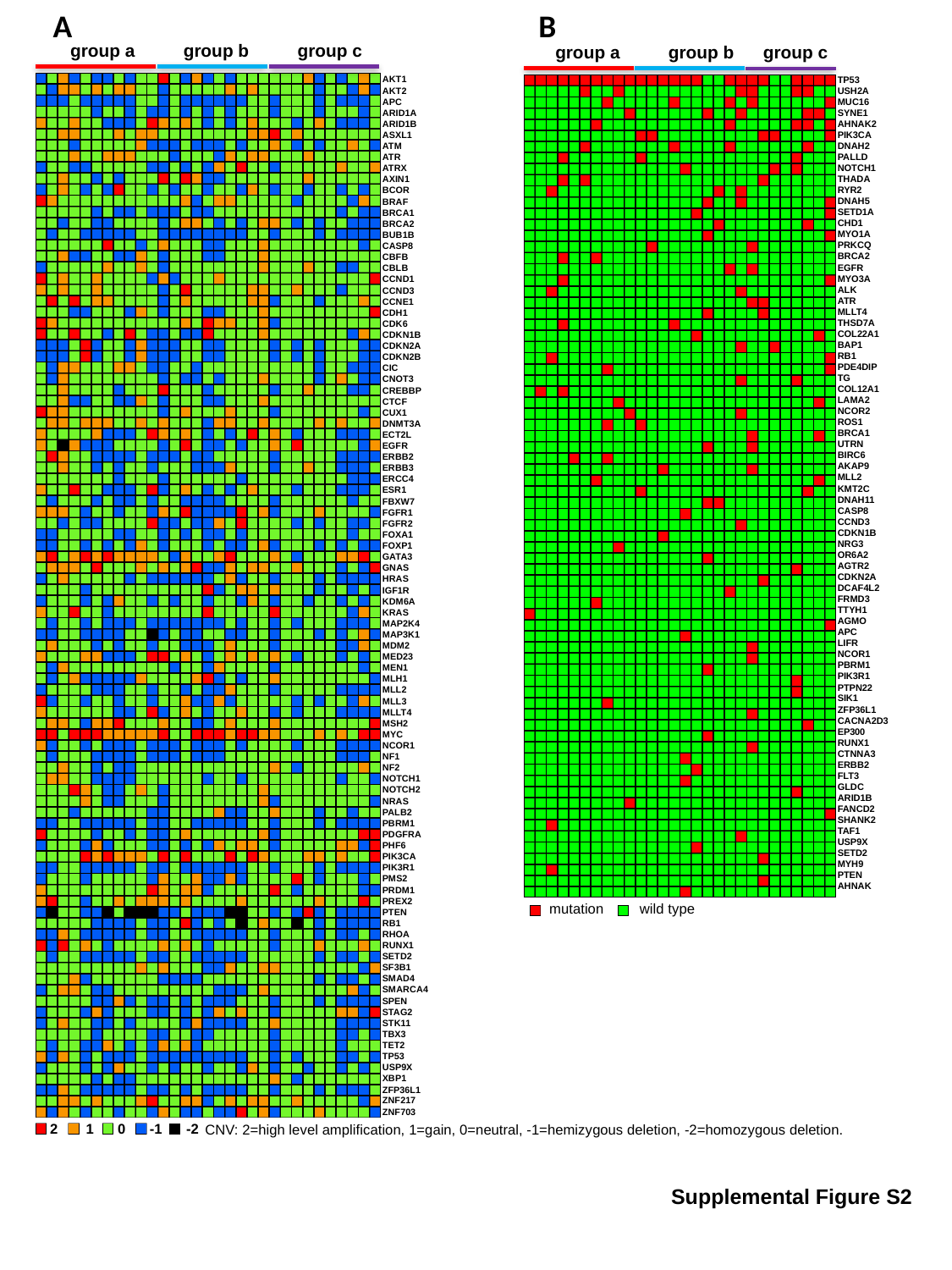

A B
group a group b group c
AKT1
AKT2
APC
ARID1A
ARID1B
ASXL1
ATM
ATR
ATRX
AXIN1
BCOR
BRAF
BRCA1
BRCA2
BUB1B
CASP8
CBFB
CBLB
CCND1
CCND3
CCNE1
CDH1
CDK6
CDKN1B
CDKN2A
CDKN2B
CIC
CNOT3
CREBBP
CTCF
CUX1
DNMT3A
ECT2L
EGFR
ERBB2
ERBB3
ERCC4
ESR1
FBXW7
FGFR1
FGFR2
FOXA1
FOXP1
GATA3
GNAS
HRAS
IGF1R
KDM6A
KRAS
MAP2K4
MAP3K1
MDM2
MED23
MEN1
MLH1
MLL2
MLL3
MLLT4
MSH2
MYC
NCOR1
NF1
NF2
NOTCH1
NOTCH2
NRAS
PALB2
PBRM1
PDGFRA
PHF6
PIK3CA
PIK3R1
PMS2
PRDM1
PREX2
PTEN
RB1
RHOA
RUNX1
SETD2
SF3B1
SMAD4
SMARCA4
SPEN
STAG2
STK11
TBX3
TET2
TP53
USP9X
XBP1
ZFP36L1
ZNF217
ZNF703
2 1 0 -1 -2
CNV: 2=high level amplification, 1=gain, 0=neutral, -1=hemizygous deletion, -2=homozygous deletion.
group a group b group c
TP53
USH2A
MUC16
SYNE1
AHNAK2
PIK3CA
DNAH2
PALLD
NOTCH1
THADA
RYR2
DNAH5
SETD1A
CHD1
MYO1A
PRKCQ
BRCA2
EGFR
MYO3A
ALK
ATR
MLLT4
THSD7A
COL22A1
BAP1
RB1
PDE4DIP
TG
COL12A1
LAMA2
NCOR2
ROS1
BRCA1
UTRN
BIRC6
AKAP9
MLL2
KMT2C
DNAH11
CASP8
CCND3
CDKN1B
NRG3
OR6A2
AGTR2
CDKN2A
DCAF4L2
FRMD3
TTYH1
AGMO
APC
LIFR
NCOR1
PBRM1
PIK3R1
PTPN22
SIK1
ZFP36L1
CACNA2D3
EP300
RUNX1
CTNNA3
ERBB2
FLT3
GLDC
ARID1B
FANCD2
SHANK2
TAF1
USP9X
SETD2
MYH9
PTEN
AHNAK
mutation wild type
Supplemental Figure S2

## Slide 4
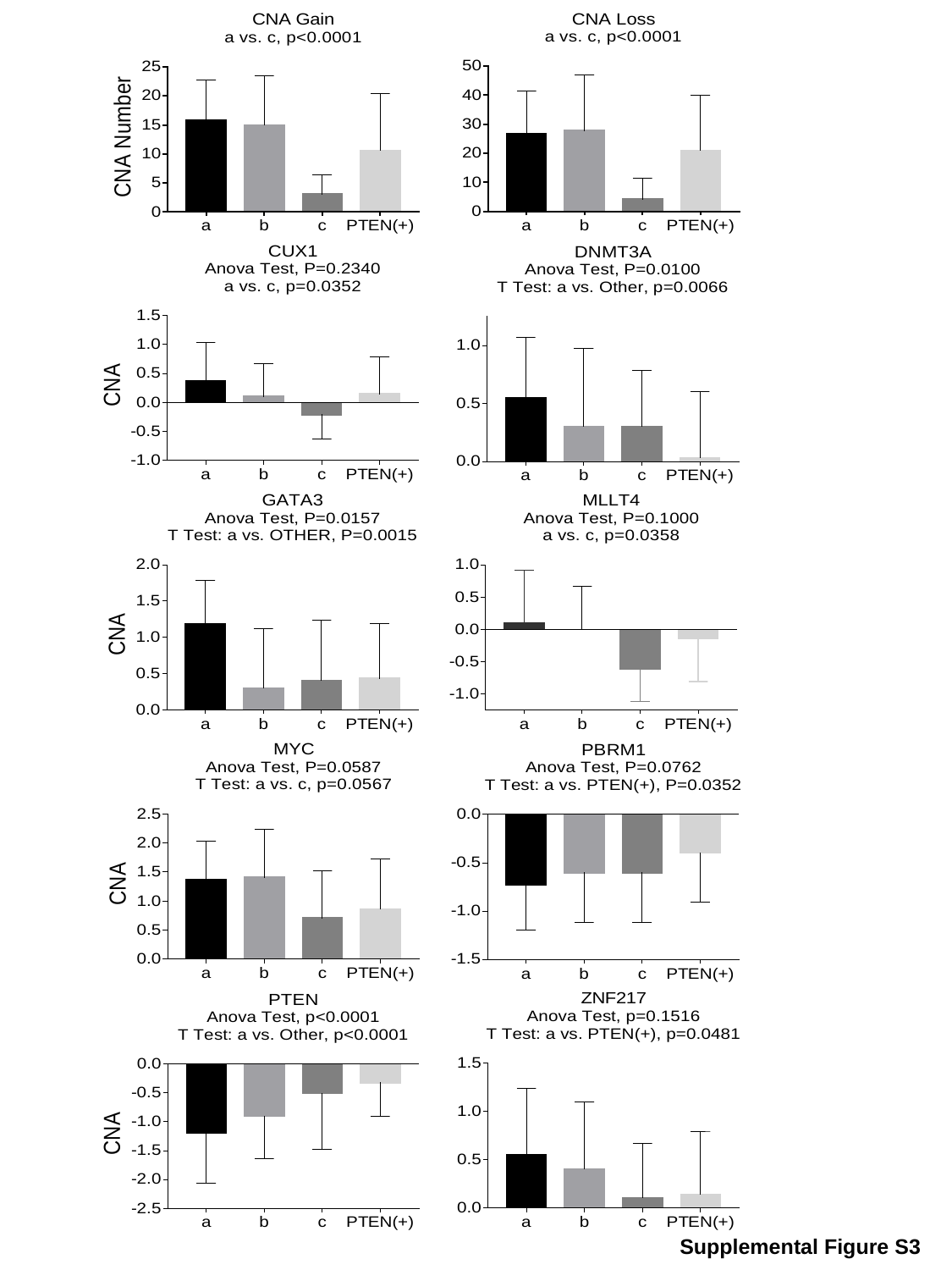

Supplemental Figure S3

## Slide 5
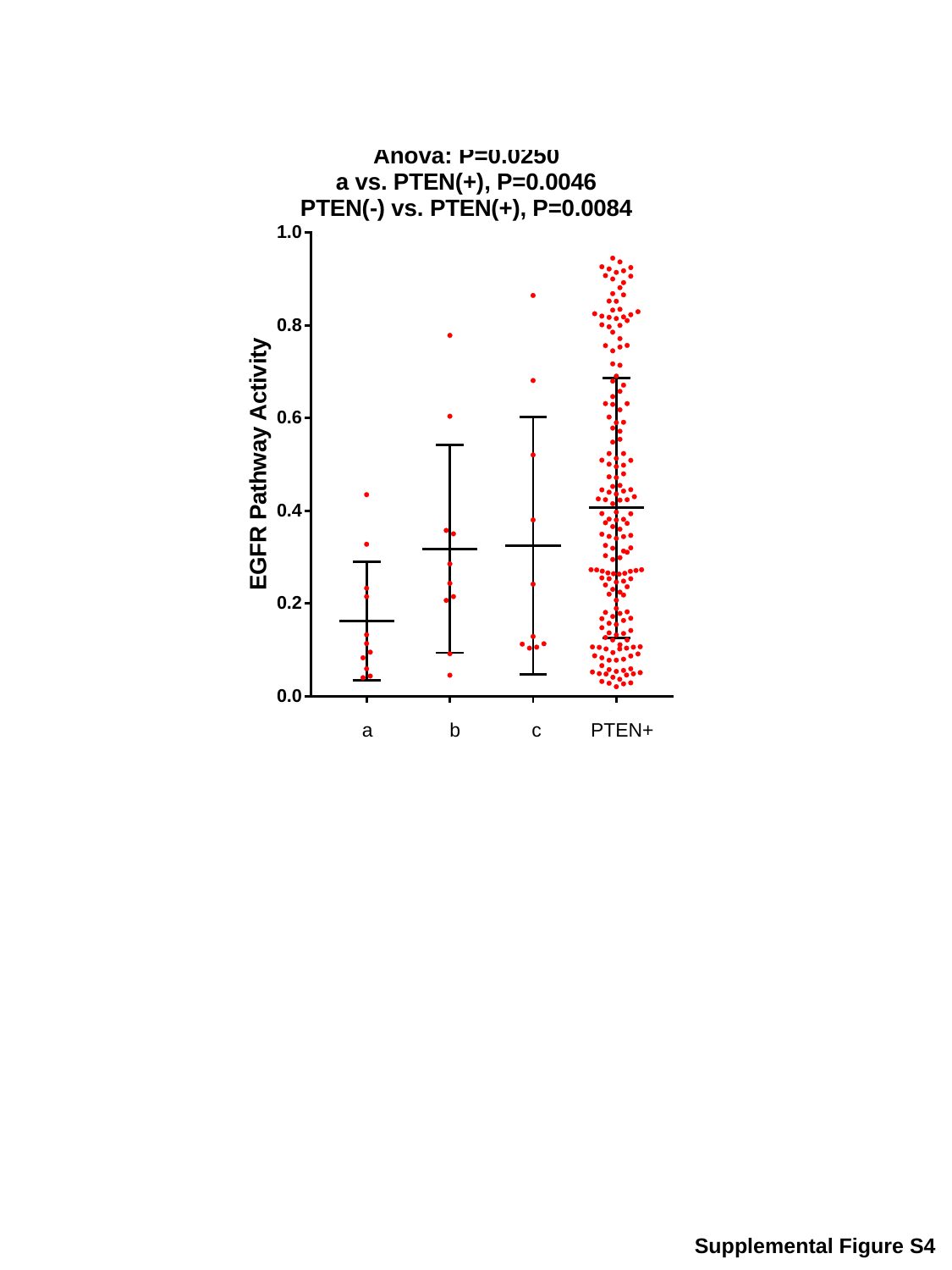

a b c PTEN+
Supplemental Figure S4

## Slide 6
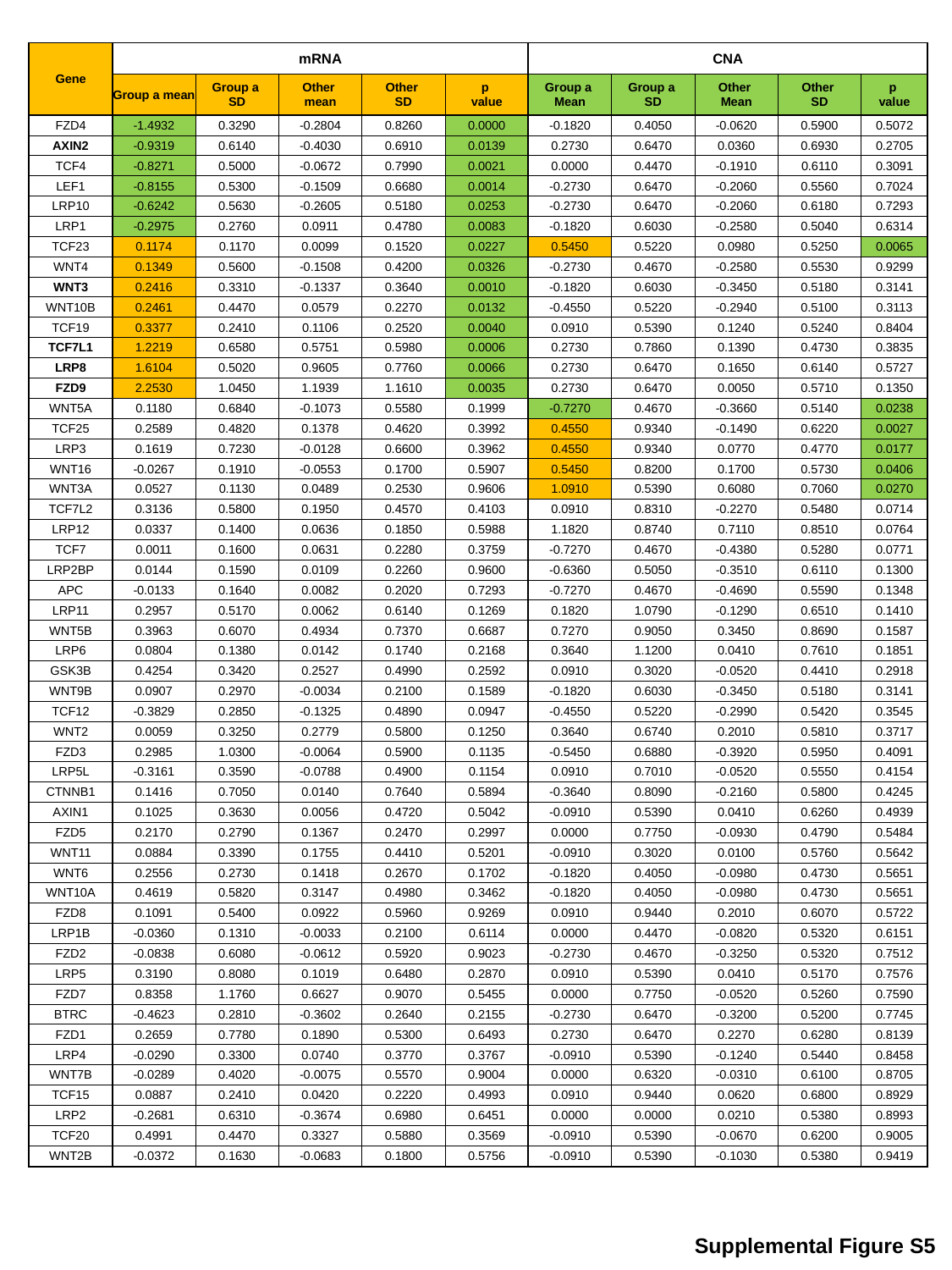

Gene expression and CNA of Wnt/β-catenin signaling
(T test: Group a vs. Other in 205TNBC)
| Gene | mRNA | | | | | CNA | | | | |
| --- | --- | --- | --- | --- | --- | --- | --- | --- | --- | --- |
| | Group a mean | Group a SD | Other mean | Other SD | p value | Group a Mean | Group a SD | Other Mean | Other SD | p value |
| FZD4 | -1.4932 | 0.3290 | -0.2804 | 0.8260 | 0.0000 | -0.1820 | 0.4050 | -0.0620 | 0.5900 | 0.5072 |
| AXIN2 | -0.9319 | 0.6140 | -0.4030 | 0.6910 | 0.0139 | 0.2730 | 0.6470 | 0.0360 | 0.6930 | 0.2705 |
| TCF4 | -0.8271 | 0.5000 | -0.0672 | 0.7990 | 0.0021 | 0.0000 | 0.4470 | -0.1910 | 0.6110 | 0.3091 |
| LEF1 | -0.8155 | 0.5300 | -0.1509 | 0.6680 | 0.0014 | -0.2730 | 0.6470 | -0.2060 | 0.5560 | 0.7024 |
| LRP10 | -0.6242 | 0.5630 | -0.2605 | 0.5180 | 0.0253 | -0.2730 | 0.6470 | -0.2060 | 0.6180 | 0.7293 |
| LRP1 | -0.2975 | 0.2760 | 0.0911 | 0.4780 | 0.0083 | -0.1820 | 0.6030 | -0.2580 | 0.5040 | 0.6314 |
| TCF23 | 0.1174 | 0.1170 | 0.0099 | 0.1520 | 0.0227 | 0.5450 | 0.5220 | 0.0980 | 0.5250 | 0.0065 |
| WNT4 | 0.1349 | 0.5600 | -0.1508 | 0.4200 | 0.0326 | -0.2730 | 0.4670 | -0.2580 | 0.5530 | 0.9299 |
| WNT3 | 0.2416 | 0.3310 | -0.1337 | 0.3640 | 0.0010 | -0.1820 | 0.6030 | -0.3450 | 0.5180 | 0.3141 |
| WNT10B | 0.2461 | 0.4470 | 0.0579 | 0.2270 | 0.0132 | -0.4550 | 0.5220 | -0.2940 | 0.5100 | 0.3113 |
| TCF19 | 0.3377 | 0.2410 | 0.1106 | 0.2520 | 0.0040 | 0.0910 | 0.5390 | 0.1240 | 0.5240 | 0.8404 |
| TCF7L1 | 1.2219 | 0.6580 | 0.5751 | 0.5980 | 0.0006 | 0.2730 | 0.7860 | 0.1390 | 0.4730 | 0.3835 |
| LRP8 | 1.6104 | 0.5020 | 0.9605 | 0.7760 | 0.0066 | 0.2730 | 0.6470 | 0.1650 | 0.6140 | 0.5727 |
| FZD9 | 2.2530 | 1.0450 | 1.1939 | 1.1610 | 0.0035 | 0.2730 | 0.6470 | 0.0050 | 0.5710 | 0.1350 |
| WNT5A | 0.1180 | 0.6840 | -0.1073 | 0.5580 | 0.1999 | -0.7270 | 0.4670 | -0.3660 | 0.5140 | 0.0238 |
| TCF25 | 0.2589 | 0.4820 | 0.1378 | 0.4620 | 0.3992 | 0.4550 | 0.9340 | -0.1490 | 0.6220 | 0.0027 |
| LRP3 | 0.1619 | 0.7230 | -0.0128 | 0.6600 | 0.3962 | 0.4550 | 0.9340 | 0.0770 | 0.4770 | 0.0177 |
| WNT16 | -0.0267 | 0.1910 | -0.0553 | 0.1700 | 0.5907 | 0.5450 | 0.8200 | 0.1700 | 0.5730 | 0.0406 |
| WNT3A | 0.0527 | 0.1130 | 0.0489 | 0.2530 | 0.9606 | 1.0910 | 0.5390 | 0.6080 | 0.7060 | 0.0270 |
| TCF7L2 | 0.3136 | 0.5800 | 0.1950 | 0.4570 | 0.4103 | 0.0910 | 0.8310 | -0.2270 | 0.5480 | 0.0714 |
| LRP12 | 0.0337 | 0.1400 | 0.0636 | 0.1850 | 0.5988 | 1.1820 | 0.8740 | 0.7110 | 0.8510 | 0.0764 |
| TCF7 | 0.0011 | 0.1600 | 0.0631 | 0.2280 | 0.3759 | -0.7270 | 0.4670 | -0.4380 | 0.5280 | 0.0771 |
| LRP2BP | 0.0144 | 0.1590 | 0.0109 | 0.2260 | 0.9600 | -0.6360 | 0.5050 | -0.3510 | 0.6110 | 0.1300 |
| APC | -0.0133 | 0.1640 | 0.0082 | 0.2020 | 0.7293 | -0.7270 | 0.4670 | -0.4690 | 0.5590 | 0.1348 |
| LRP11 | 0.2957 | 0.5170 | 0.0062 | 0.6140 | 0.1269 | 0.1820 | 1.0790 | -0.1290 | 0.6510 | 0.1410 |
| WNT5B | 0.3963 | 0.6070 | 0.4934 | 0.7370 | 0.6687 | 0.7270 | 0.9050 | 0.3450 | 0.8690 | 0.1587 |
| LRP6 | 0.0804 | 0.1380 | 0.0142 | 0.1740 | 0.2168 | 0.3640 | 1.1200 | 0.0410 | 0.7610 | 0.1851 |
| GSK3B | 0.4254 | 0.3420 | 0.2527 | 0.4990 | 0.2592 | 0.0910 | 0.3020 | -0.0520 | 0.4410 | 0.2918 |
| WNT9B | 0.0907 | 0.2970 | -0.0034 | 0.2100 | 0.1589 | -0.1820 | 0.6030 | -0.3450 | 0.5180 | 0.3141 |
| TCF12 | -0.3829 | 0.2850 | -0.1325 | 0.4890 | 0.0947 | -0.4550 | 0.5220 | -0.2990 | 0.5420 | 0.3545 |
| WNT2 | 0.0059 | 0.3250 | 0.2779 | 0.5800 | 0.1250 | 0.3640 | 0.6740 | 0.2010 | 0.5810 | 0.3717 |
| FZD3 | 0.2985 | 1.0300 | -0.0064 | 0.5900 | 0.1135 | -0.5450 | 0.6880 | -0.3920 | 0.5950 | 0.4091 |
| LRP5L | -0.3161 | 0.3590 | -0.0788 | 0.4900 | 0.1154 | 0.0910 | 0.7010 | -0.0520 | 0.5550 | 0.4154 |
| CTNNB1 | 0.1416 | 0.7050 | 0.0140 | 0.7640 | 0.5894 | -0.3640 | 0.8090 | -0.2160 | 0.5800 | 0.4245 |
| AXIN1 | 0.1025 | 0.3630 | 0.0056 | 0.4720 | 0.5042 | -0.0910 | 0.5390 | 0.0410 | 0.6260 | 0.4939 |
| FZD5 | 0.2170 | 0.2790 | 0.1367 | 0.2470 | 0.2997 | 0.0000 | 0.7750 | -0.0930 | 0.4790 | 0.5484 |
| WNT11 | 0.0884 | 0.3390 | 0.1755 | 0.4410 | 0.5201 | -0.0910 | 0.3020 | 0.0100 | 0.5760 | 0.5642 |
| WNT6 | 0.2556 | 0.2730 | 0.1418 | 0.2670 | 0.1702 | -0.1820 | 0.4050 | -0.0980 | 0.4730 | 0.5651 |
| WNT10A | 0.4619 | 0.5820 | 0.3147 | 0.4980 | 0.3462 | -0.1820 | 0.4050 | -0.0980 | 0.4730 | 0.5651 |
| FZD8 | 0.1091 | 0.5400 | 0.0922 | 0.5960 | 0.9269 | 0.0910 | 0.9440 | 0.2010 | 0.6070 | 0.5722 |
| LRP1B | -0.0360 | 0.1310 | -0.0033 | 0.2100 | 0.6114 | 0.0000 | 0.4470 | -0.0820 | 0.5320 | 0.6151 |
| FZD2 | -0.0838 | 0.6080 | -0.0612 | 0.5920 | 0.9023 | -0.2730 | 0.4670 | -0.3250 | 0.5320 | 0.7512 |
| LRP5 | 0.3190 | 0.8080 | 0.1019 | 0.6480 | 0.2870 | 0.0910 | 0.5390 | 0.0410 | 0.5170 | 0.7576 |
| FZD7 | 0.8358 | 1.1760 | 0.6627 | 0.9070 | 0.5455 | 0.0000 | 0.7750 | -0.0520 | 0.5260 | 0.7590 |
| BTRC | -0.4623 | 0.2810 | -0.3602 | 0.2640 | 0.2155 | -0.2730 | 0.6470 | -0.3200 | 0.5200 | 0.7745 |
| FZD1 | 0.2659 | 0.7780 | 0.1890 | 0.5300 | 0.6493 | 0.2730 | 0.6470 | 0.2270 | 0.6280 | 0.8139 |
| LRP4 | -0.0290 | 0.3300 | 0.0740 | 0.3770 | 0.3767 | -0.0910 | 0.5390 | -0.1240 | 0.5440 | 0.8458 |
| WNT7B | -0.0289 | 0.4020 | -0.0075 | 0.5570 | 0.9004 | 0.0000 | 0.6320 | -0.0310 | 0.6100 | 0.8705 |
| TCF15 | 0.0887 | 0.2410 | 0.0420 | 0.2220 | 0.4993 | 0.0910 | 0.9440 | 0.0620 | 0.6800 | 0.8929 |
| LRP2 | -0.2681 | 0.6310 | -0.3674 | 0.6980 | 0.6451 | 0.0000 | 0.0000 | 0.0210 | 0.5380 | 0.8993 |
| TCF20 | 0.4991 | 0.4470 | 0.3327 | 0.5880 | 0.3569 | -0.0910 | 0.5390 | -0.0670 | 0.6200 | 0.9005 |
| WNT2B | -0.0372 | 0.1630 | -0.0683 | 0.1800 | 0.5756 | -0.0910 | 0.5390 | -0.1030 | 0.5380 | 0.9419 |
Supplemental Figure S5

## Slide 7
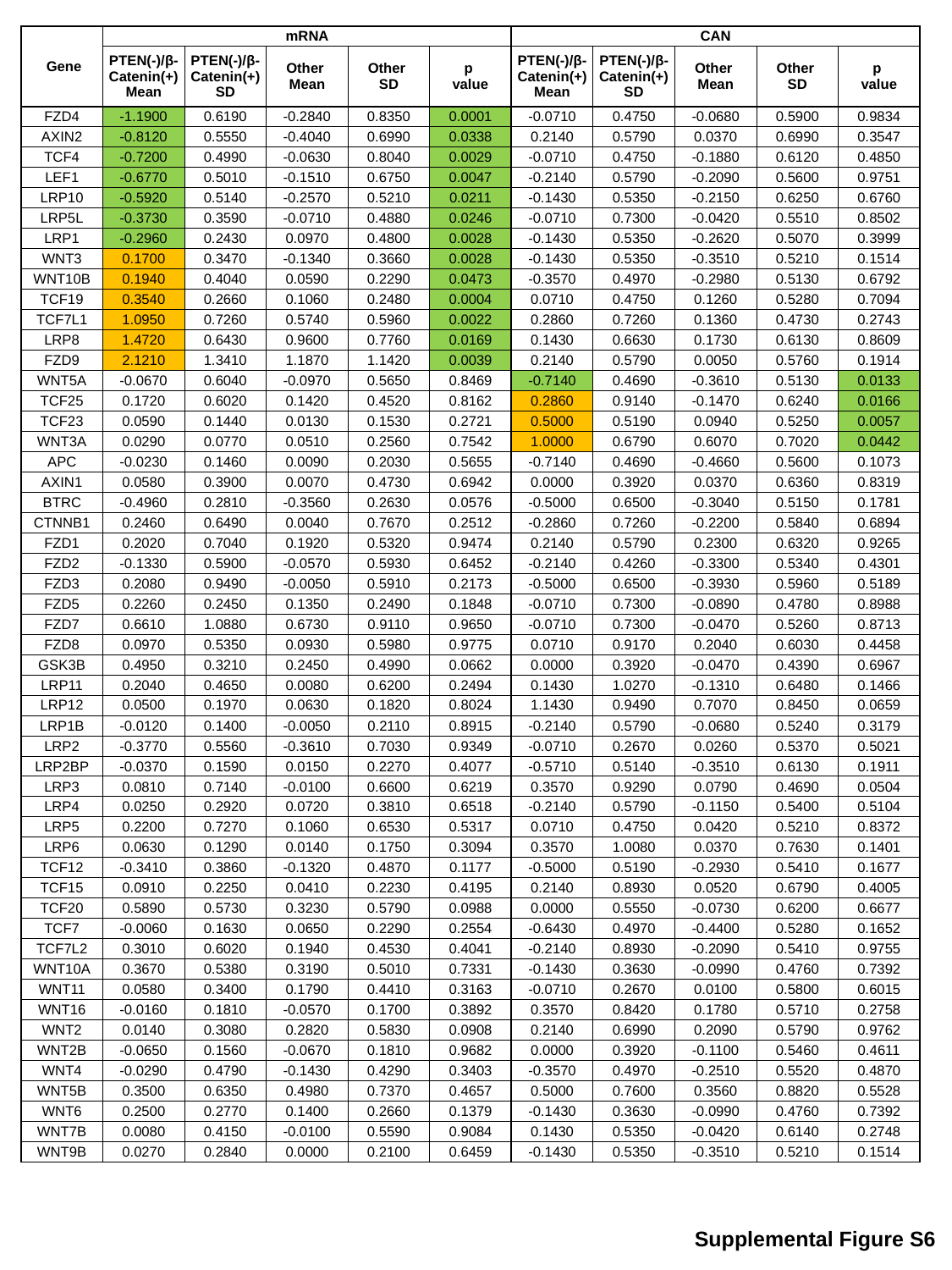

Gene expression and CNA of Wnt/β-catenin signaling
(T test: PTEN(-)/β-catenin(+) vs. Other in 205TNBC)
| Gene | mRNA | | | | | CAN | | | | |
| --- | --- | --- | --- | --- | --- | --- | --- | --- | --- | --- |
| | PTEN(-)/β-Catenin(+) Mean | PTEN(-)/β-Catenin(+) SD | Other Mean | Other SD | p value | PTEN(-)/β-Catenin(+) Mean | PTEN(-)/β-Catenin(+) SD | Other Mean | Other SD | p value |
| FZD4 | -1.1900 | 0.6190 | -0.2840 | 0.8350 | 0.0001 | -0.0710 | 0.4750 | -0.0680 | 0.5900 | 0.9834 |
| AXIN2 | -0.8120 | 0.5550 | -0.4040 | 0.6990 | 0.0338 | 0.2140 | 0.5790 | 0.0370 | 0.6990 | 0.3547 |
| TCF4 | -0.7200 | 0.4990 | -0.0630 | 0.8040 | 0.0029 | -0.0710 | 0.4750 | -0.1880 | 0.6120 | 0.4850 |
| LEF1 | -0.6770 | 0.5010 | -0.1510 | 0.6750 | 0.0047 | -0.2140 | 0.5790 | -0.2090 | 0.5600 | 0.9751 |
| LRP10 | -0.5920 | 0.5140 | -0.2570 | 0.5210 | 0.0211 | -0.1430 | 0.5350 | -0.2150 | 0.6250 | 0.6760 |
| LRP5L | -0.3730 | 0.3590 | -0.0710 | 0.4880 | 0.0246 | -0.0710 | 0.7300 | -0.0420 | 0.5510 | 0.8502 |
| LRP1 | -0.2960 | 0.2430 | 0.0970 | 0.4800 | 0.0028 | -0.1430 | 0.5350 | -0.2620 | 0.5070 | 0.3999 |
| WNT3 | 0.1700 | 0.3470 | -0.1340 | 0.3660 | 0.0028 | -0.1430 | 0.5350 | -0.3510 | 0.5210 | 0.1514 |
| WNT10B | 0.1940 | 0.4040 | 0.0590 | 0.2290 | 0.0473 | -0.3570 | 0.4970 | -0.2980 | 0.5130 | 0.6792 |
| TCF19 | 0.3540 | 0.2660 | 0.1060 | 0.2480 | 0.0004 | 0.0710 | 0.4750 | 0.1260 | 0.5280 | 0.7094 |
| TCF7L1 | 1.0950 | 0.7260 | 0.5740 | 0.5960 | 0.0022 | 0.2860 | 0.7260 | 0.1360 | 0.4730 | 0.2743 |
| LRP8 | 1.4720 | 0.6430 | 0.9600 | 0.7760 | 0.0169 | 0.1430 | 0.6630 | 0.1730 | 0.6130 | 0.8609 |
| FZD9 | 2.1210 | 1.3410 | 1.1870 | 1.1420 | 0.0039 | 0.2140 | 0.5790 | 0.0050 | 0.5760 | 0.1914 |
| WNT5A | -0.0670 | 0.6040 | -0.0970 | 0.5650 | 0.8469 | -0.7140 | 0.4690 | -0.3610 | 0.5130 | 0.0133 |
| TCF25 | 0.1720 | 0.6020 | 0.1420 | 0.4520 | 0.8162 | 0.2860 | 0.9140 | -0.1470 | 0.6240 | 0.0166 |
| TCF23 | 0.0590 | 0.1440 | 0.0130 | 0.1530 | 0.2721 | 0.5000 | 0.5190 | 0.0940 | 0.5250 | 0.0057 |
| WNT3A | 0.0290 | 0.0770 | 0.0510 | 0.2560 | 0.7542 | 1.0000 | 0.6790 | 0.6070 | 0.7020 | 0.0442 |
| APC | -0.0230 | 0.1460 | 0.0090 | 0.2030 | 0.5655 | -0.7140 | 0.4690 | -0.4660 | 0.5600 | 0.1073 |
| AXIN1 | 0.0580 | 0.3900 | 0.0070 | 0.4730 | 0.6942 | 0.0000 | 0.3920 | 0.0370 | 0.6360 | 0.8319 |
| BTRC | -0.4960 | 0.2810 | -0.3560 | 0.2630 | 0.0576 | -0.5000 | 0.6500 | -0.3040 | 0.5150 | 0.1781 |
| CTNNB1 | 0.2460 | 0.6490 | 0.0040 | 0.7670 | 0.2512 | -0.2860 | 0.7260 | -0.2200 | 0.5840 | 0.6894 |
| FZD1 | 0.2020 | 0.7040 | 0.1920 | 0.5320 | 0.9474 | 0.2140 | 0.5790 | 0.2300 | 0.6320 | 0.9265 |
| FZD2 | -0.1330 | 0.5900 | -0.0570 | 0.5930 | 0.6452 | -0.2140 | 0.4260 | -0.3300 | 0.5340 | 0.4301 |
| FZD3 | 0.2080 | 0.9490 | -0.0050 | 0.5910 | 0.2173 | -0.5000 | 0.6500 | -0.3930 | 0.5960 | 0.5189 |
| FZD5 | 0.2260 | 0.2450 | 0.1350 | 0.2490 | 0.1848 | -0.0710 | 0.7300 | -0.0890 | 0.4780 | 0.8988 |
| FZD7 | 0.6610 | 1.0880 | 0.6730 | 0.9110 | 0.9650 | -0.0710 | 0.7300 | -0.0470 | 0.5260 | 0.8713 |
| FZD8 | 0.0970 | 0.5350 | 0.0930 | 0.5980 | 0.9775 | 0.0710 | 0.9170 | 0.2040 | 0.6030 | 0.4458 |
| GSK3B | 0.4950 | 0.3210 | 0.2450 | 0.4990 | 0.0662 | 0.0000 | 0.3920 | -0.0470 | 0.4390 | 0.6967 |
| LRP11 | 0.2040 | 0.4650 | 0.0080 | 0.6200 | 0.2494 | 0.1430 | 1.0270 | -0.1310 | 0.6480 | 0.1466 |
| LRP12 | 0.0500 | 0.1970 | 0.0630 | 0.1820 | 0.8024 | 1.1430 | 0.9490 | 0.7070 | 0.8450 | 0.0659 |
| LRP1B | -0.0120 | 0.1400 | -0.0050 | 0.2110 | 0.8915 | -0.2140 | 0.5790 | -0.0680 | 0.5240 | 0.3179 |
| LRP2 | -0.3770 | 0.5560 | -0.3610 | 0.7030 | 0.9349 | -0.0710 | 0.2670 | 0.0260 | 0.5370 | 0.5021 |
| LRP2BP | -0.0370 | 0.1590 | 0.0150 | 0.2270 | 0.4077 | -0.5710 | 0.5140 | -0.3510 | 0.6130 | 0.1911 |
| LRP3 | 0.0810 | 0.7140 | -0.0100 | 0.6600 | 0.6219 | 0.3570 | 0.9290 | 0.0790 | 0.4690 | 0.0504 |
| LRP4 | 0.0250 | 0.2920 | 0.0720 | 0.3810 | 0.6518 | -0.2140 | 0.5790 | -0.1150 | 0.5400 | 0.5104 |
| LRP5 | 0.2200 | 0.7270 | 0.1060 | 0.6530 | 0.5317 | 0.0710 | 0.4750 | 0.0420 | 0.5210 | 0.8372 |
| LRP6 | 0.0630 | 0.1290 | 0.0140 | 0.1750 | 0.3094 | 0.3570 | 1.0080 | 0.0370 | 0.7630 | 0.1401 |
| TCF12 | -0.3410 | 0.3860 | -0.1320 | 0.4870 | 0.1177 | -0.5000 | 0.5190 | -0.2930 | 0.5410 | 0.1677 |
| TCF15 | 0.0910 | 0.2250 | 0.0410 | 0.2230 | 0.4195 | 0.2140 | 0.8930 | 0.0520 | 0.6790 | 0.4005 |
| TCF20 | 0.5890 | 0.5730 | 0.3230 | 0.5790 | 0.0988 | 0.0000 | 0.5550 | -0.0730 | 0.6200 | 0.6677 |
| TCF7 | -0.0060 | 0.1630 | 0.0650 | 0.2290 | 0.2554 | -0.6430 | 0.4970 | -0.4400 | 0.5280 | 0.1652 |
| TCF7L2 | 0.3010 | 0.6020 | 0.1940 | 0.4530 | 0.4041 | -0.2140 | 0.8930 | -0.2090 | 0.5410 | 0.9755 |
| WNT10A | 0.3670 | 0.5380 | 0.3190 | 0.5010 | 0.7331 | -0.1430 | 0.3630 | -0.0990 | 0.4760 | 0.7392 |
| WNT11 | 0.0580 | 0.3400 | 0.1790 | 0.4410 | 0.3163 | -0.0710 | 0.2670 | 0.0100 | 0.5800 | 0.6015 |
| WNT16 | -0.0160 | 0.1810 | -0.0570 | 0.1700 | 0.3892 | 0.3570 | 0.8420 | 0.1780 | 0.5710 | 0.2758 |
| WNT2 | 0.0140 | 0.3080 | 0.2820 | 0.5830 | 0.0908 | 0.2140 | 0.6990 | 0.2090 | 0.5790 | 0.9762 |
| WNT2B | -0.0650 | 0.1560 | -0.0670 | 0.1810 | 0.9682 | 0.0000 | 0.3920 | -0.1100 | 0.5460 | 0.4611 |
| WNT4 | -0.0290 | 0.4790 | -0.1430 | 0.4290 | 0.3403 | -0.3570 | 0.4970 | -0.2510 | 0.5520 | 0.4870 |
| WNT5B | 0.3500 | 0.6350 | 0.4980 | 0.7370 | 0.4657 | 0.5000 | 0.7600 | 0.3560 | 0.8820 | 0.5528 |
| WNT6 | 0.2500 | 0.2770 | 0.1400 | 0.2660 | 0.1379 | -0.1430 | 0.3630 | -0.0990 | 0.4760 | 0.7392 |
| WNT7B | 0.0080 | 0.4150 | -0.0100 | 0.5590 | 0.9084 | 0.1430 | 0.5350 | -0.0420 | 0.6140 | 0.2748 |
| WNT9B | 0.0270 | 0.2840 | 0.0000 | 0.2100 | 0.6459 | -0.1430 | 0.5350 | -0.3510 | 0.5210 | 0.1514 |
Supplemental Figure S6

## Slide 8
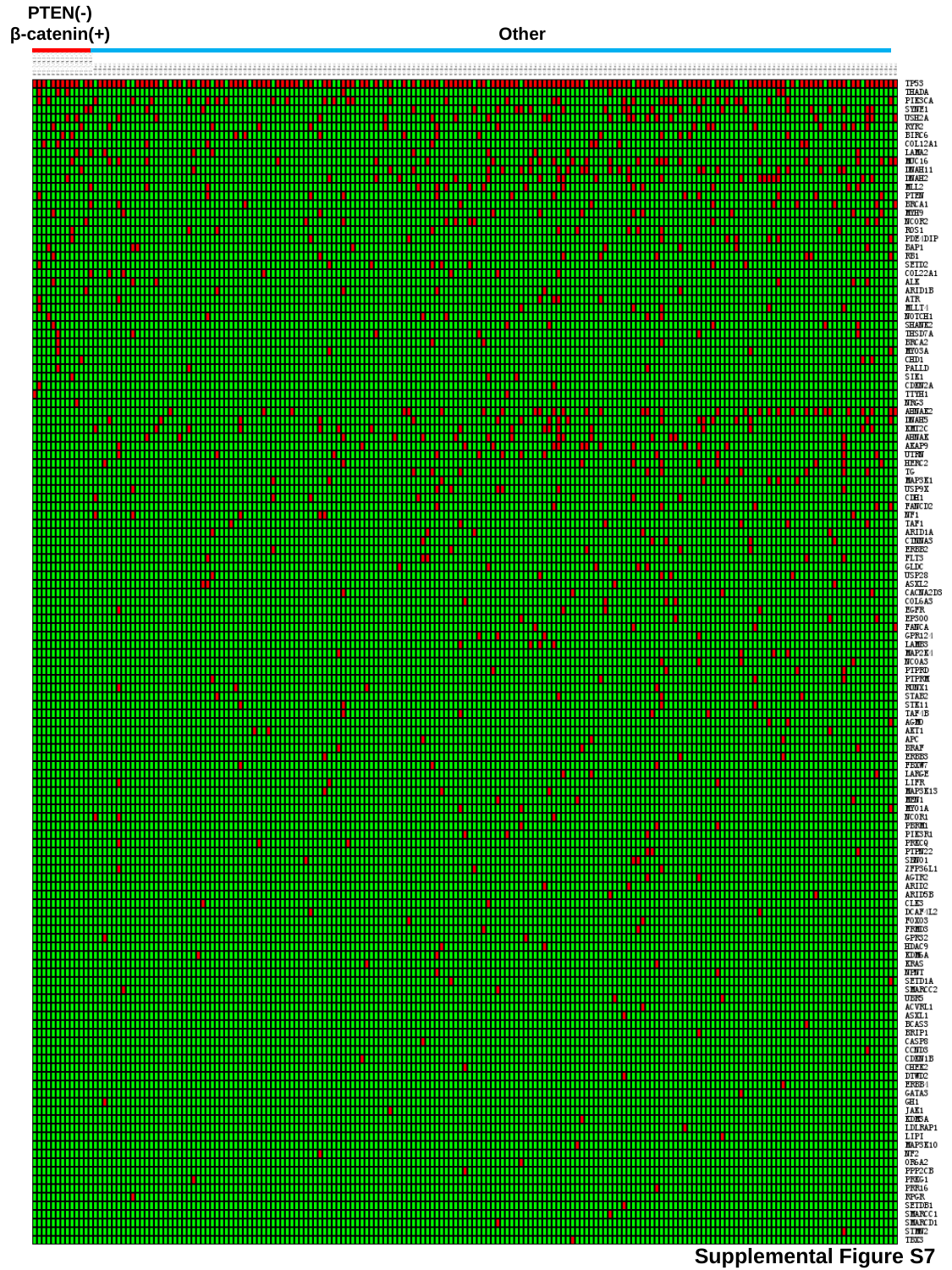

Gene mutation in PTEN/β-catenin groups
PTEN(-)
β-catenin(+)
Other
Supplemental Figure S7

## Slide 9
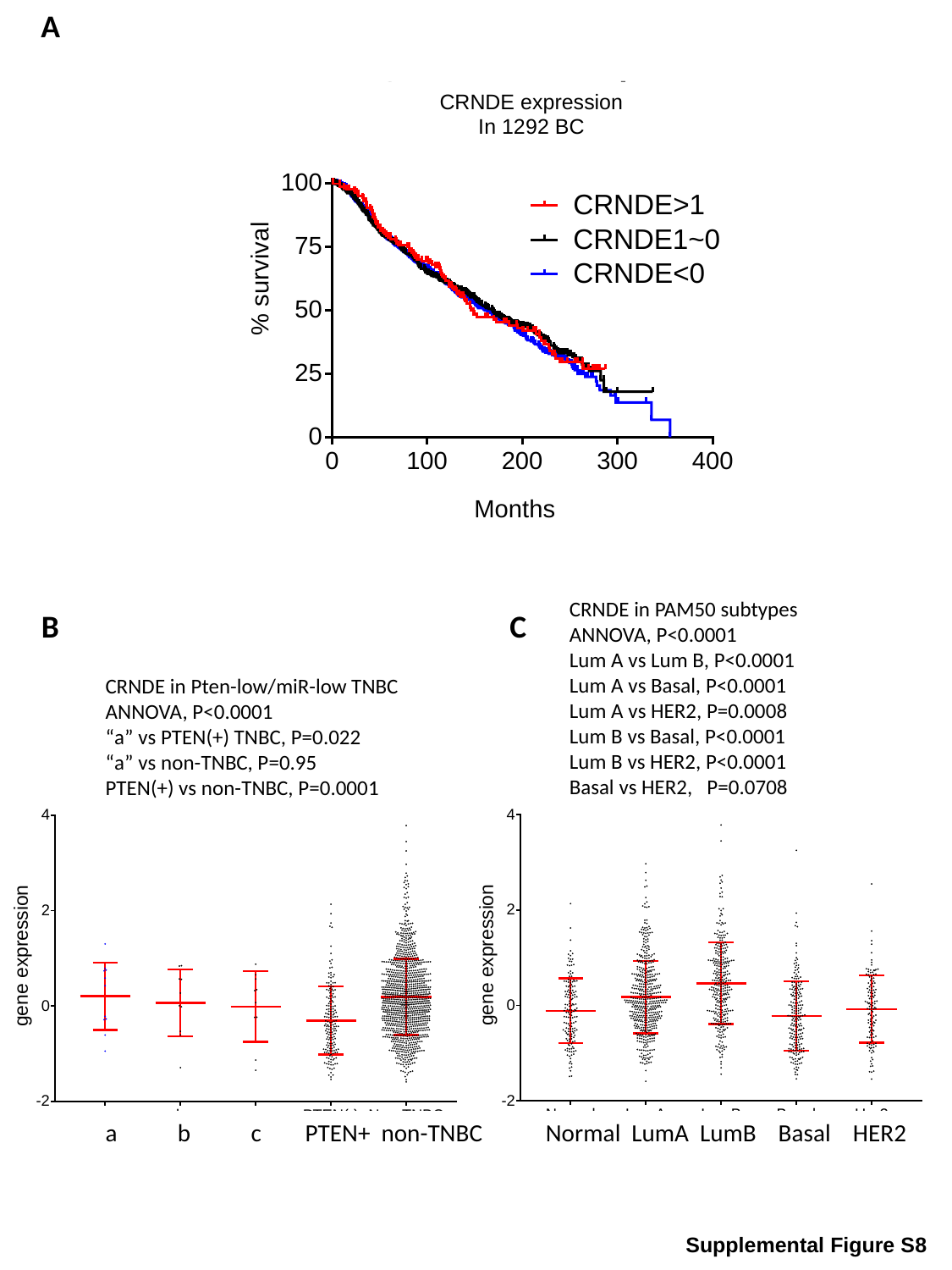

A
CRNDE expression in 5miRs groups
and PAM50 subtypes
CRNDE expression
In 1292 BC
 % survival
Months
CRNDE in PAM50 subtypes
ANNOVA, P<0.0001
Lum A vs Lum B, P<0.0001
Lum A vs Basal, P<0.0001
Lum A vs HER2, P=0.0008
Lum B vs Basal, P<0.0001
Lum B vs HER2, P<0.0001
Basal vs HER2, P=0.0708
B
C
CRNDE in Pten-low/miR-low TNBC
ANNOVA, P<0.0001
“a” vs PTEN(+) TNBC, P=0.022
“a” vs non-TNBC, P=0.95
PTEN(+) vs non-TNBC, P=0.0001
a b c PTEN+ non-TNBC
Normal LumA LumB Basal HER2
Supplemental Figure S8

## Slide 10
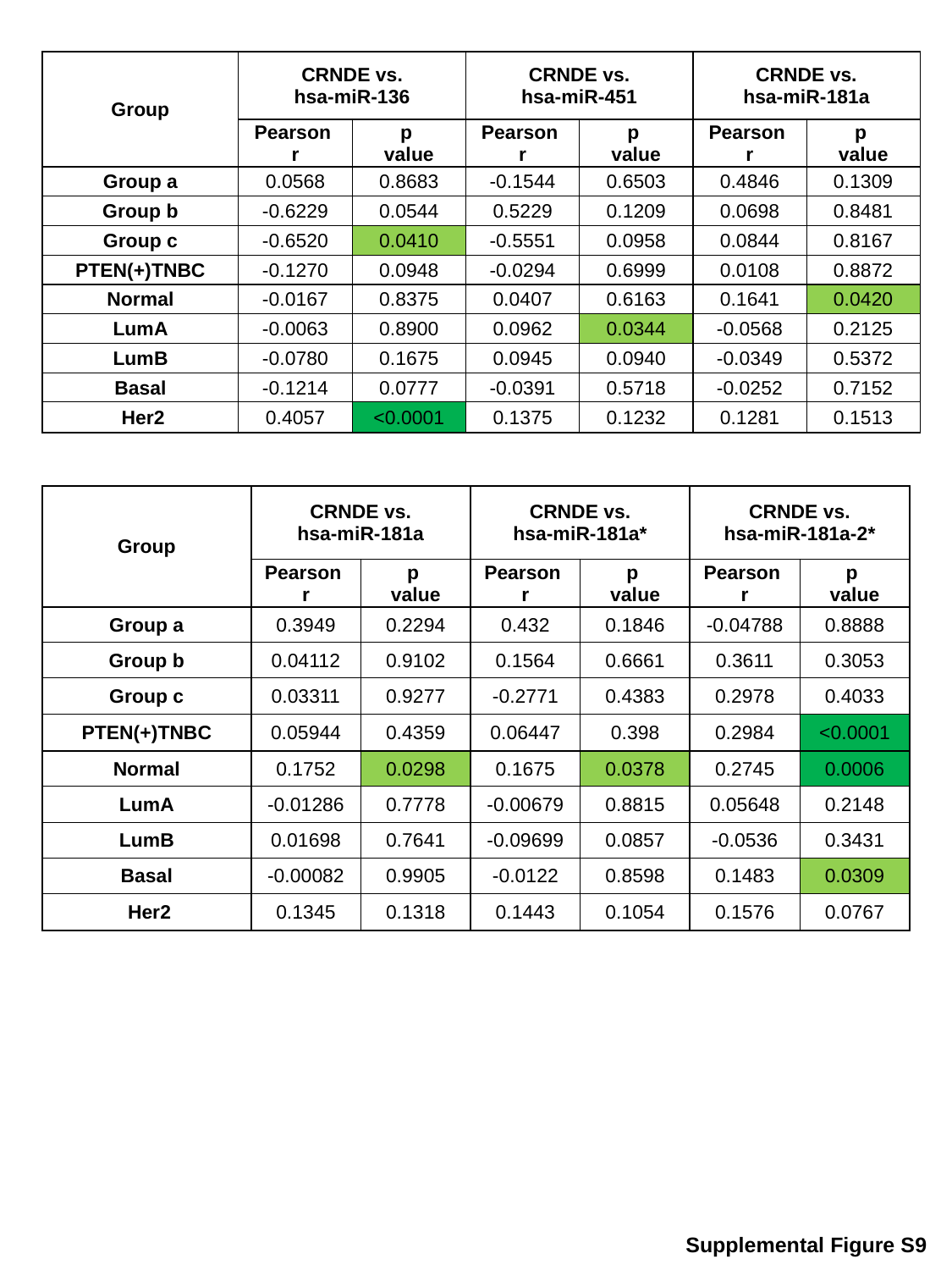

Correlation between CRNDE mRNA and targeted
miR-136, miR-451 and 4 miR-181a-related miRNAs
in 5miRs groups and PAM50 subtypes
| Group | CRNDE vs. hsa-miR-136 | | CRNDE vs. hsa-miR-451 | | CRNDE vs. hsa-miR-181a | |
| --- | --- | --- | --- | --- | --- | --- |
| | Pearson r | p value | Pearson r | p value | Pearson r | p value |
| Group a | 0.0568 | 0.8683 | -0.1544 | 0.6503 | 0.4846 | 0.1309 |
| Group b | -0.6229 | 0.0544 | 0.5229 | 0.1209 | 0.0698 | 0.8481 |
| Group c | -0.6520 | 0.0410 | -0.5551 | 0.0958 | 0.0844 | 0.8167 |
| PTEN(+)TNBC | -0.1270 | 0.0948 | -0.0294 | 0.6999 | 0.0108 | 0.8872 |
| Normal | -0.0167 | 0.8375 | 0.0407 | 0.6163 | 0.1641 | 0.0420 |
| LumA | -0.0063 | 0.8900 | 0.0962 | 0.0344 | -0.0568 | 0.2125 |
| LumB | -0.0780 | 0.1675 | 0.0945 | 0.0940 | -0.0349 | 0.5372 |
| Basal | -0.1214 | 0.0777 | -0.0391 | 0.5718 | -0.0252 | 0.7152 |
| Her2 | 0.4057 | <0.0001 | 0.1375 | 0.1232 | 0.1281 | 0.1513 |
| Group | CRNDE vs. hsa-miR-181a | | CRNDE vs. hsa-miR-181a\* | | CRNDE vs. hsa-miR-181a-2\* | |
| --- | --- | --- | --- | --- | --- | --- |
| | Pearson r | p value | Pearson r | p value | Pearson r | p value |
| Group a | 0.3949 | 0.2294 | 0.432 | 0.1846 | -0.04788 | 0.8888 |
| Group b | 0.04112 | 0.9102 | 0.1564 | 0.6661 | 0.3611 | 0.3053 |
| Group c | 0.03311 | 0.9277 | -0.2771 | 0.4383 | 0.2978 | 0.4033 |
| PTEN(+)TNBC | 0.05944 | 0.4359 | 0.06447 | 0.398 | 0.2984 | <0.0001 |
| Normal | 0.1752 | 0.0298 | 0.1675 | 0.0378 | 0.2745 | 0.0006 |
| LumA | -0.01286 | 0.7778 | -0.00679 | 0.8815 | 0.05648 | 0.2148 |
| LumB | 0.01698 | 0.7641 | -0.09699 | 0.0857 | -0.0536 | 0.3431 |
| Basal | -0.00082 | 0.9905 | -0.0122 | 0.8598 | 0.1483 | 0.0309 |
| Her2 | 0.1345 | 0.1318 | 0.1443 | 0.1054 | 0.1576 | 0.0767 |
Supplemental Figure S9

## Slide 11
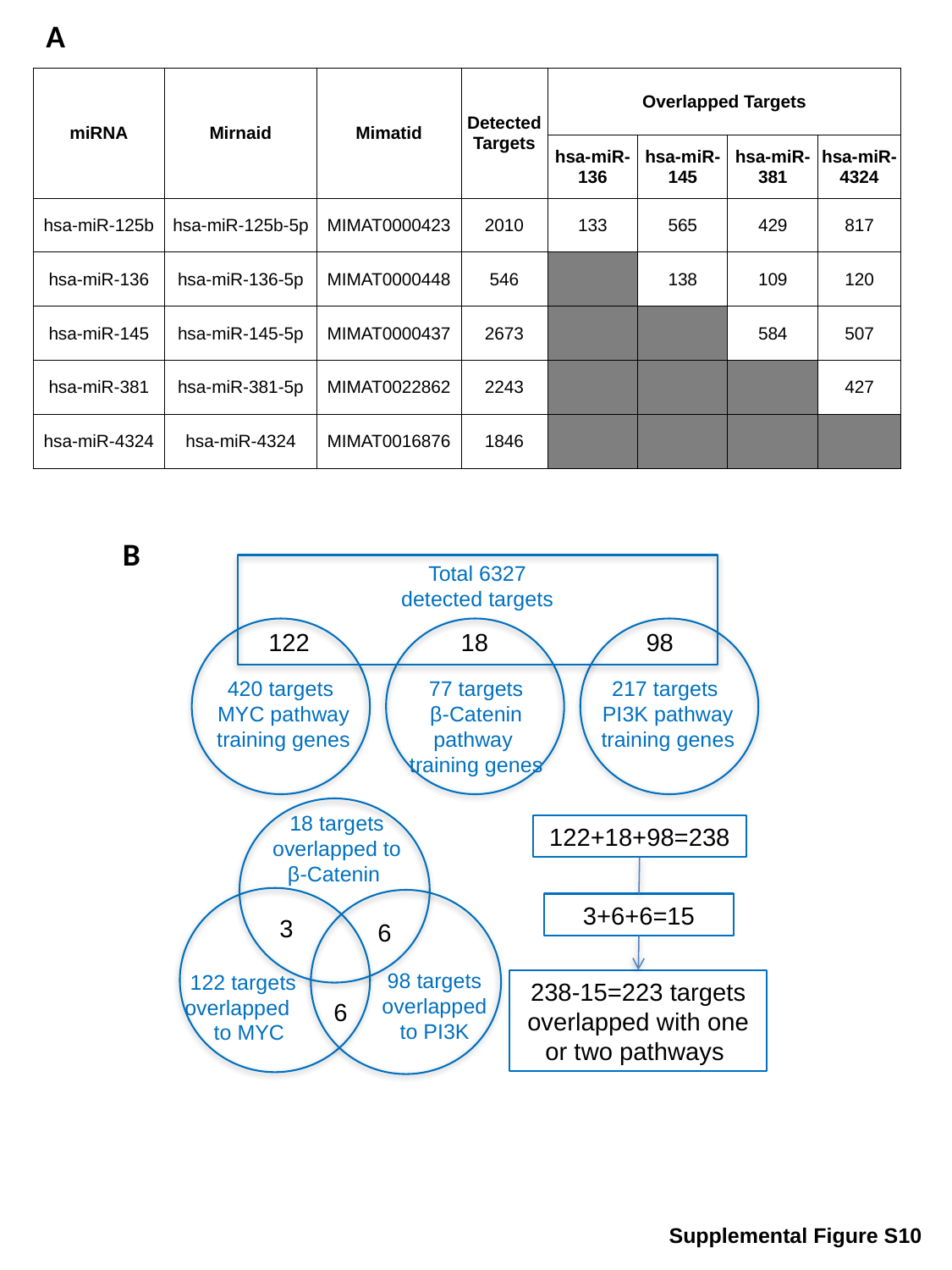

A
| miRNA | Mirnaid | Mimatid | Detected Targets | Overlapped Targets | | | |
| --- | --- | --- | --- | --- | --- | --- | --- |
| | | | | hsa-miR-136 | hsa-miR-145 | hsa-miR-381 | hsa-miR-4324 |
| hsa-miR-125b | hsa-miR-125b-5p | MIMAT0000423 | 2010 | 133 | 565 | 429 | 817 |
| hsa-miR-136 | hsa-miR-136-5p | MIMAT0000448 | 546 | | 138 | 109 | 120 |
| hsa-miR-145 | hsa-miR-145-5p | MIMAT0000437 | 2673 | | | 584 | 507 |
| hsa-miR-381 | hsa-miR-381-5p | MIMAT0022862 | 2243 | | | | 427 |
| hsa-miR-4324 | hsa-miR-4324 | MIMAT0016876 | 1846 | | | | |
B
Total 6327
detected targets
122
18
98
217 targets
PI3K pathway
training genes
420 targets
MYC pathway
training genes
77 targets
β-Catenin pathway
training genes
18 targets overlapped to β-Catenin
3
6
98 targets overlapped to PI3K
122 targets overlapped
 to MYC
6
122+18+98=238
3+6+6=15
238-15=223 targets overlapped with one or two pathways
Supplemental Figure S10

## Slide 12
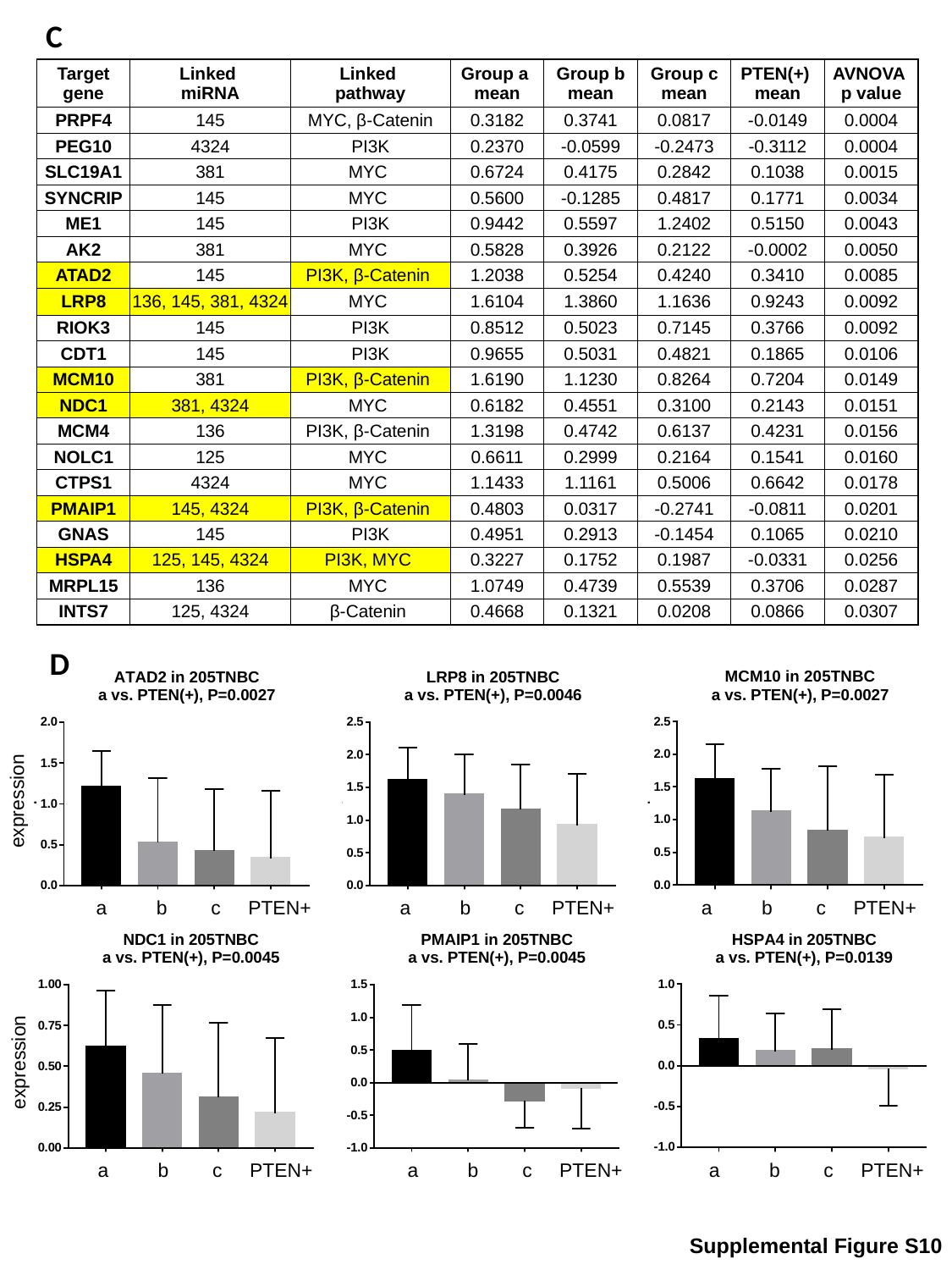

C
| Target gene | Linked miRNA | Linked pathway | Group a mean | Group b mean | Group c mean | PTEN(+) mean | AVNOVA p value |
| --- | --- | --- | --- | --- | --- | --- | --- |
| PRPF4 | 145 | MYC, β-Catenin | 0.3182 | 0.3741 | 0.0817 | -0.0149 | 0.0004 |
| PEG10 | 4324 | PI3K | 0.2370 | -0.0599 | -0.2473 | -0.3112 | 0.0004 |
| SLC19A1 | 381 | MYC | 0.6724 | 0.4175 | 0.2842 | 0.1038 | 0.0015 |
| SYNCRIP | 145 | MYC | 0.5600 | -0.1285 | 0.4817 | 0.1771 | 0.0034 |
| ME1 | 145 | PI3K | 0.9442 | 0.5597 | 1.2402 | 0.5150 | 0.0043 |
| AK2 | 381 | MYC | 0.5828 | 0.3926 | 0.2122 | -0.0002 | 0.0050 |
| ATAD2 | 145 | PI3K, β-Catenin | 1.2038 | 0.5254 | 0.4240 | 0.3410 | 0.0085 |
| LRP8 | 136, 145, 381, 4324 | MYC | 1.6104 | 1.3860 | 1.1636 | 0.9243 | 0.0092 |
| RIOK3 | 145 | PI3K | 0.8512 | 0.5023 | 0.7145 | 0.3766 | 0.0092 |
| CDT1 | 145 | PI3K | 0.9655 | 0.5031 | 0.4821 | 0.1865 | 0.0106 |
| MCM10 | 381 | PI3K, β-Catenin | 1.6190 | 1.1230 | 0.8264 | 0.7204 | 0.0149 |
| NDC1 | 381, 4324 | MYC | 0.6182 | 0.4551 | 0.3100 | 0.2143 | 0.0151 |
| MCM4 | 136 | PI3K, β-Catenin | 1.3198 | 0.4742 | 0.6137 | 0.4231 | 0.0156 |
| NOLC1 | 125 | MYC | 0.6611 | 0.2999 | 0.2164 | 0.1541 | 0.0160 |
| CTPS1 | 4324 | MYC | 1.1433 | 1.1161 | 0.5006 | 0.6642 | 0.0178 |
| PMAIP1 | 145, 4324 | PI3K, β-Catenin | 0.4803 | 0.0317 | -0.2741 | -0.0811 | 0.0201 |
| GNAS | 145 | PI3K | 0.4951 | 0.2913 | -0.1454 | 0.1065 | 0.0210 |
| HSPA4 | 125, 145, 4324 | PI3K, MYC | 0.3227 | 0.1752 | 0.1987 | -0.0331 | 0.0256 |
| MRPL15 | 136 | MYC | 1.0749 | 0.4739 | 0.5539 | 0.3706 | 0.0287 |
| INTS7 | 125, 4324 | β-Catenin | 0.4668 | 0.1321 | 0.0208 | 0.0866 | 0.0307 |
D
expression
a b c PTEN+
a b c PTEN+
a b c PTEN+
expression
a b c PTEN+
a b c PTEN+
a b c PTEN+
Supplemental Figure S10

## Slide 13
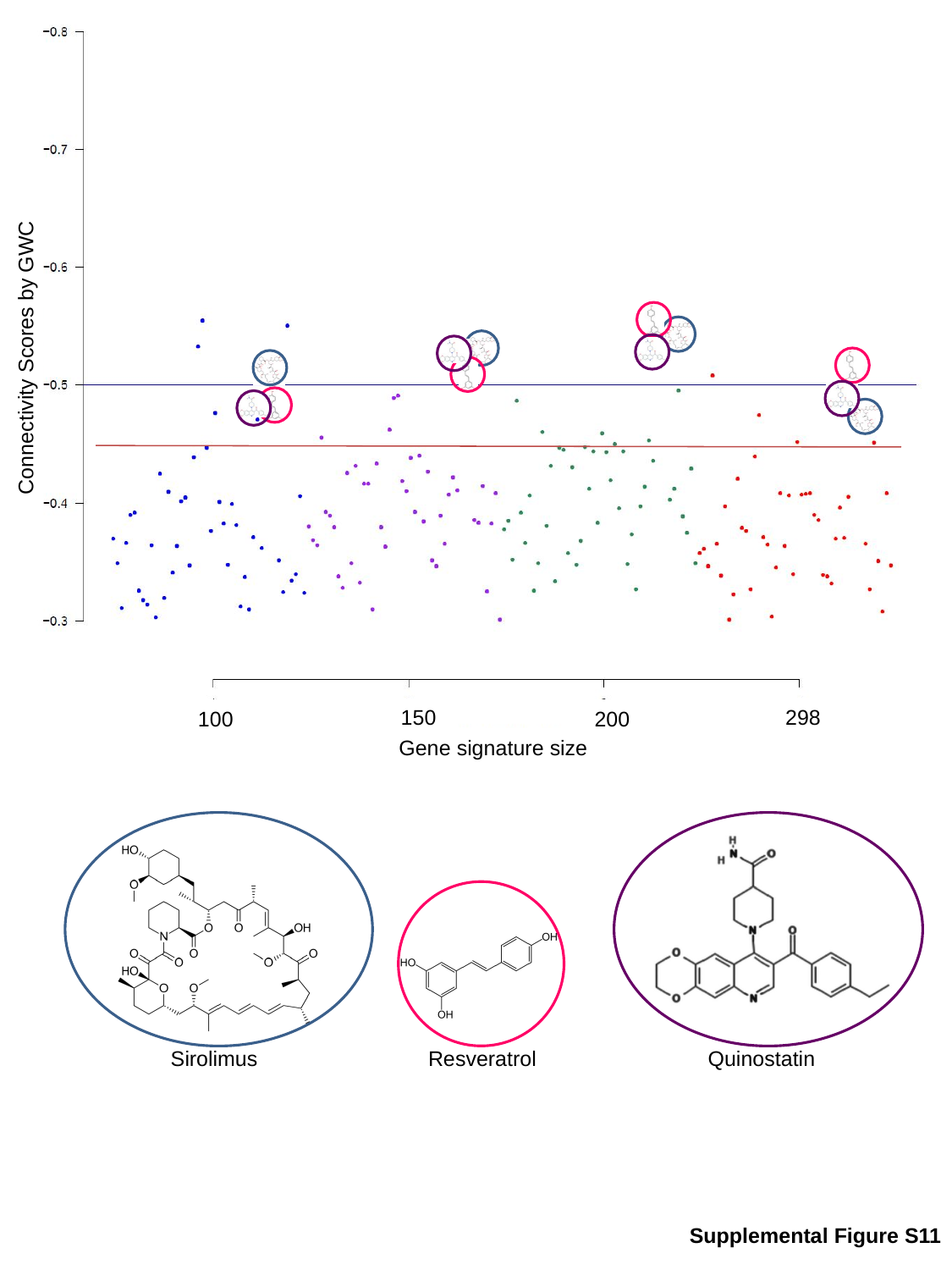

Connectivity Scores by GWC
298
150
100
200
Gene signature size
Sirolimus
Resveratrol
Quinostatin
Supplemental Figure S11

## Slide 14
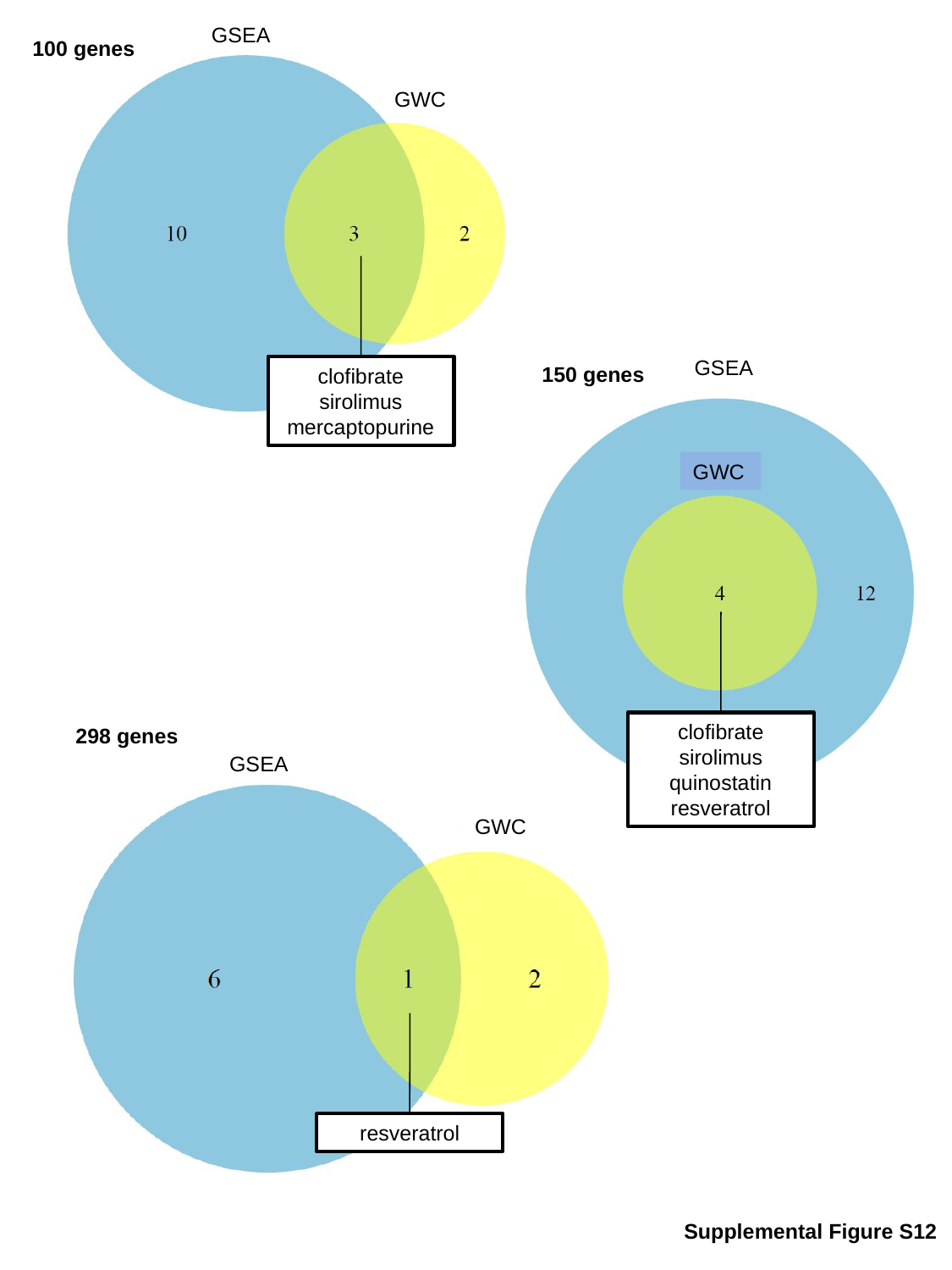

GSEA
100 genes
GWC
GSEA
150 genes
clofibrate
sirolimus
mercaptopurine
GWC
clofibrate
sirolimus
quinostatin
resveratrol
298 genes
GSEA
GWC
resveratrol
Supplemental Figure S12
